# A radial histogenetic model of the mouse pallial amygdala

**DOI:** 10.1007/s00429-020-02097-4

**Published:** 2020-06-24

**Authors:** Elena Garcia-Calero, Margaret Martínez-de-la-Torre, Luis Puelles

**Affiliations:** Department of Human Anatomy, School of Medicine and IMIB-Arrixaca Institute, University of Murcia, 30120 Murcia, Spain

**Keywords:** Histogenesis, Radial migration, Stratification, Amygdalar pallium, Amygdalar structure, Amygdalar nuclei

## Abstract

Conventional anatomic models of the rodent (mammalian) amygdala are based on section planes oblique to its intrinsic radial glial organization. As a result, we still lack a model of amygdalar histogenesis in terms of radial units (progenitor domains and related radial migration and layering patterns). A radial model of the mouse pallial amygdala is first offered here, based on three logical steps: (1) analysis of amygdalar radial structure in variously discriminative genoarchitectonic material, using an optimal *ad hoc* section plane; (2) testing preliminary models with experiments labelling at the brain surface single packets of radial glia processes, to be followed into the ventricular surface across intervening predicted elements; (3) selection of 81 differential amygdalar gene markers and checking planar and radial aspects of their distribution across the model elements. This approach shows that subtle changes to the conventional schema of the amygdala allow a radial histogenetic model to be recognized, which is consistent with molecularly coded differential identities of its units and strata. It is expected that this model will help both causal studies of amygdalar developmental patterning and comparative evolutionary studies. It also may have potential impact on hodological and functional studies.

**Electronic supplementary material:**

The online version of this article (10.1007/s00429-020-02097-4) contains supplementary material, which is available to authorized users.

## Introduction

The amygdala encloses a heterogeneous group of nuclear masses found at the tip of the temporal lobe. This complex is involved in evaluating potential environmental danger and consequent emotional processing, notably fear (Swanson and Petrovich 1998; Sah et al. 2003; Phelps and Ledoux 2005; Ledoux 2007; Whalen and Phelps 2009; Rolls 2015; Olucha-Bordonau et al. 2015; Medina et al. 2017). Amygdalar nuclei were conventionally classified according to cyto- and myeloarchitectonic characteristics, topography and functional significance (Johnston et al. 1923; Brockhaus 1938; Gastaut and Lammers 1961; Krettek and Price 1978; Turner and Zimmer 1984; Price et al. 1987; Alheid et al. 1995; Gloor 1997; Swanson and Petrovich 1998; McDonald 2003; De Olmos et al. 2004; Ledoux 2007; Martinez-García et al. 2008, 2012; Olucha-Bordonau et al. 2015). Other studies investigated histochemical/immunocytochemical aspects or neurotransmitter characteristics (e.g., Cassel et al. 1986; McDonald and Augustine 1993; Sun and Cassell 1993; Swanson and Petrovich 1998; Kemppainen and Pitkänen 2000; Berdel and Morys 2000; Legaz et al. 2005). Molecular patterning studies of the amygdala analysed various gene expression patterns and some relevant loss-of-function phenotypes in the mouse, though this topic is still in its infancy (Puelles et al. 2000; Stenman et al. 2003; Remedios et al. 2004, 2007; Tole et al. 2005; Garcia-Lopez et al. 2008; Cocas et al. 2011; Tang et al. 2012; Silberberg et al. 2016; Puelles et al. 2016a). Various approaches led to distinction of pallial and subpallial amygdalar regions (Johnston et al. 1923; Holmgren 1925; Swanson and Petrovich 1998; Puelles et al. 2000; Medina et al. 2004; García-Lopez et al. 2008; Olucha-Bordonau et al. 2015; Puelles et al. 2016a; Medina et al. 2017). The pallial amygdala is actually topologically *external* to the cortical pallium, building a self-contained histogenetic macro-complex in connection with the hypothalamus (Puelles et al. 2019).

Evolutionary changes in overall pallial morphogenesis among mammals caused different degrees of anteroposterior rotation of the temporal pole around the central insular area, changing the appearance of amygdalar elements in coronal sections. In primates there occurs also latero-medial rotation of the temporal pole, affecting likewise the topography of the amygdala. There is nevertheless a wide consensus on a fundamental schema of amygdalar partitions shared among all mammals (Johnston 1923; Olucha-Bordonau et al. 2015; Medina et al. 2017).

We are not aware of attempts to classify pallial amygdalar components into *radial histogenetic units*, that is, to systematize the standard set of nuclei as stratified derivatives of distinct ventricular progenitor domains. This endeavour represents our main line of interest in this report. Due to the variety of pallial amygdalar nuclei recognized so far, it may be expected that they arise from several amygdalar progenitor domains. However, we do not know how many progenitor domains or radial units constitute the pallial amygdala, nor their spatial arrangement in three dimensions. Some known amygdalar nuclei obviously are deep periventricular elements, as observed in standard atlases, whereas others clearly are superficial. Our understanding of intermediate amygdalar entities is most sketchy. Curiously, apparently intermediate structures do not agree in number with either deep or superficial ones. Accordingly, we may be missing some amygdalar components, which possibly have been lumped within the classic concepts.

The molecular era has changed the classical strategy of attending mainly to apparent topography and cell typology in neuroanatomic analysis. Now we also are interested in *causal explanations* of observed structure, related to developmental and evolutionary understanding (Nieuwenhuys and Puelles 2016). Neural histogenetic phenomena first obey two-dimensional molecular patterning of neuroepithelial progenitor fields. Such regionalization phenomena rely on obtaining differential genomic readout based on the different distance of progenitor cells relative to various sources of signalling morphogens (across criss-crossing gradients of diffused signals). The progenitors are provided with molecular tool kits enabling them to react with differential genomic read-outs to local concentrations of given morphogens. Resulting activation/inactivation of downstream genes across neighbouring neuroepithelial areas creates ‘delimited distinct progenitor domains’ having singular molecular profiles. This is *molecular regionalization*, or patterning, the precursor of future *anatomic subdivision*. In the histogenetic phase of development (proliferation, neurogenesis, migration, and stratification) said early molecular patterning guides differential neuroepithelial proliferation, neurogenesis and gliogenesis, leading to diversified regional cell populations and emergent anatomic fates.

Our study examines the radial units that build the mouse pallial amygdala. This knowledge should illuminate histogenetic and positional relationships bearing on causal developmental patterning mechanisms, so far wholly unknown for the amygdala. We postulated that it should be possible to group parsimoniously the well-known amygdalar pallial nuclei into radial complexes across periventricular, intermediate and superficial strata, after attending to appropriate section planes and radial glial structure. Since the mouse amygdalar ventricular surface lies in a *coronal* plane caudally to most of the amygdala, while the amygdalar pial surface is *horizontal*, the radial glial processes interconnecting these two surfaces trace curves approximating an obliquity of 45° (Remedios et al. 2007). The study of amygdalar radial glial structure was accordingly another goal addressed here, both descriptively and experimentally. An oblique *radial amygdalar* section plane was, therefore, used *ad hoc* as a new approach in the study of pallial amygdalar radial complexes. It was further expected that true radial units might show unique molecular profiles, irrespective of a number of shared determinants. Analysis of such differential molecular characteristics was a further aim of our study.

Our molecular description is based on adult AZIN2-LacZ labelling, combined with a glimpse at *Lhx9* and *Er81* expression (Nery et al. 2002; Remedios et al. 2004; Tole et al. 2005; Garcia-Lopez et al. 2008; Abellán et al. 2009; Tosches et al. 2018). The AZIN2 gene (ornithine-decarboxylase antizyme inhibitor 2) codes a non-functional ornithine-decarboxylase paralog protein, which blocks specific antizymes (AZ1-3), thus increasing polyamine synthesis and function (López-Garcia et al. 2013). It is heterogeneously present in the adult mouse brain (Martínez-de-la-Torre et al. 2018; Puelles et al. 2019), and throws new light upon adult amygdalar radial structure. This analysis provides a first notion of potential molecularly distinctive radial units stretching from the ventricle to the pial surface.

The observed radial relationships were checked in the perinatal mouse by DiI labelling at a variety of superficial points. This approach via perinatal data owes to the unreliability of immunochemical tracing of radial glia cells at older postnatal stages. DiI label applied in the fixed brains at the pial surface (staining the pial glial endfeet) was transported along the radial glial processes relative to intermediate and periventricular amygdalar subdivisions, and onto the amygdalar ventricular surface (radial glia cell bodies). This aided the elaboration of a consistent radial model of mouse pallial amygdalar structure after repeated testing of our initial conjectures.

Hoping that sets of genes expressed in this area would group consistently with the model, we examined 81 gene patterns empirically determined to be expressed differentially in the perinatal mouse amygdala (Allen Mouse Brain Atlas; Allen Developing Mouse Brain Atlas). Tabular analysis of these data in various ways validated our model.

## Materials and methods

### Animal preparation and tissue analysis

Dams showing a vaginal plug were classified as at embryonic day (*E*) 0.5. Preceding sacrifice, the embryos/perinatal animals were anesthetized on ice. For adult animals, after standard sodium pentobarbital anesthesia, the mice were perfused with 4% paraformaldehyde. The brains were dissected out and fixed overnight in 4% paraformaldehyde in pH 7.4 phosphate-buffered saline (PBS) at 4 °C. The brains were embedded in 4% agarose in PBS and 100 µm sections were cut in horizontal, sagittal, standard coronal, or amygdalar radial planes with a Leica vibratome (VT1000 S) to be processed for in situ hybridization and immunohistochemistry.

### In situ hybridization

The hybridization protocol used was according to Shimamura et al. (1994). For the riboprobe preparations we used restriction enzymes and polymerases in the presence of digoxigenin-11-UTP. Mouse cDNA probes used for in situ hybridization analysis included *Lhx9* and *Cyp26b* (our own collection) and *Er81* (S. Martínez).

### Immunohistochemistry

For immunostaining we followed protocols published in Ferran et al. (2015) and Garcia-Calero and Scharff (2013). The primary antibodies used in this study were: mouse anti-RC2 (1:10; Developmental Studies Hybridoma Bank, Iowa City, IA), mouse anti-TH (1:1000; Novus Biologicals), mouse anti-parvalbumin (1:2000; Sigma-Aldrich), rabbit anti-Calretinin (1:1500, SWANT). Some sections were counterstained with neutral red.

### Graphic projection map of superficial amygdalar structures

We obtained a graphic projection map of superficial amygdalar nuclei and surrounding areas. For this we used delineations and stereotaxic references from “The Mouse Brain in Stereotaxic Coordinates” (Paxinos and Franklin 2001). We projected graphically the borders of nuclear complexes visible in each coronal atlas slice onto a two-dimensional linear representation projected upon the horizontal stereotaxic baseline. The resulting set of lines was aligned with separations according to the relevant stereotaxic data, producing the required flat map. The distance between lines was indicated in millimeters.

### DiI experiments

Embryonic brains at E18.5 or P0 were exposed by separation of the skin and partial opening of the skull. Immediately they were fixed by immersion in 4% paraformaldehyde in pH 7.4 PBS at 4 °C during 24 h. The following day the brains were extracted from the skull, cleaned, and prepared for labelling by gentle application of filter paper to the surface to eliminate excess fluid. Under the operating microscope, a small DiI crystal (1,1′-dioctadecyl 3,3,3′,3′-tetramethyl-indocarbocyanine perchlorate; Molecular Probes) was inserted by means of a sharp tungsten needle into a point of the amygdalar pial surface selected from the graphical projection map. Thereafter the brain was returned to the fixing solution, and the dye was allowed to diffuse at 37 °C during 20–30 days in the dark. After this time, the brains were embedded and oriented for sectioning in a 4% low-melting-point agarose block, which was cut on a vibratome into 120 µm sections; these were counterstained for 30 min with DAPI (1/1000; Sigma). This material was analyzed and documented photographically using a Leica confocal microscope (details below).

### Transgenic mice

We used adult brains of a heterozygotic mice line developed at the Department of Biochemistry, School of Medicine, University of Murcia (López-Garcia et al. 2013). These mice express recombinant beta-galactosidase protein under control of the AZIN2 promoter. After standard perfusion and embedding in 4% agarose, Vibratome 120 µm thick serial sections were obtained, using the three standard section planes, as well as a special amygdalar radial plane oriented between 45° and 30° relative to an ideal plane tangent to the entorhinal cortex at the back of the brain. Floating sections were then reacted for beta-galactosidase (López-Garcia et al. 2013). Alternate series of sections often received afterwards diverse counterstains by immunoreaction against parvalbumin (PV), calretinin (CR) or tyrosine hydroxylase (TH). The latter reactions also were performed on floating sections according to the protocols reported in Ferran et al. (2015), and were finally washed, dehydrated, mounted on slides and covered.

### Allen Brain Atlas gene selection

AGEA data-mining software found as an option in the Allen Mouse Brain Atlas was used to find genes expressed non-ubiquitously in the mouse pallial amygdala (https://mouse.brain-map.org/agea). A crosshair marker was placed in various amygdalar regions recognized as containing distinct radial elements in our model, and the tool *Find genes* was used to obtain a list of candidate genes of interest. We checked in detail the expression pattern of any promising candidates, selecting only those genes (*n* = 81) which seemed sufficiently discriminative, generally showing a sharp expression pattern. The positive versus negative expression of all chosen genes across all our pallial amygdalar subdivisions was independently entered into provisional Excel sheets by two of the authors (EGC, LP). After detailed discussion of eventual differences, a consensus primary map was produced (Suppl. Table 1). To extract significance from this material we designed “expression similarity maps” referred to each of the deep, intermediate and superficial strata of the whole set of radial units postulated in the model. These maps implied simple reordering of the gene-lines corresponding to genes expressed in each amygdalar stratum within the Excel sheet (normally a subset of the whole list of markers), so that the varying degrees of sharedness found across the radial units (i.e., similarity versus dissimilarity) was graphically emphasized, thus making such differences observable at a glance (Tables 1, 2, 3). Ulteriorly we developed as well “unit-wide homogeneity maps”, in which we emphasized by an added color mark those primary data which correspond to genes which are expressed *throughout* radial domains (i.e., across all strata; Table 4). Given that a good number of genes showed ‘unit-wide homogeneity’ simultaneously in several radial units, this analysis was refined in the same Table by additional color coding, emphasizing selectively those genes which characterized homogeneously all strata of a *single* radial unit (i.e., maximal selectivity).

### Image capture, manipulation and figure assembly

Digital microphotographs were acquired using Aperio CS2 technology, and we also used a confocal microscope (Leica TCS SP8 AOBS, Leica Microsystems GmbH, Mannheim, Germany). The images (*z*-stacks) were acquired with LCS software. The digital confocal images were processed with ImageJ (NIH, https://rsb.info.nih.gov/ij), Adobe Photoshop and Adobe Illustrator software (Adobe Systems MountainView, CA,USA) and Aperio ImageScope software (Leica Microsystems GmbH, Mannheim, Germany).

## Results

### Glial structure of pallial amygdala and the radial section plane.

To explore radial histogenetic units in the mouse pallial amygdala, we first analysed expression of the radial glia marker RC2 in sagittal sections through the mouse amygdalar region and during development (E12.5–P4), with emphasis on perinatal stages. We will illustrate only results for stages E16.5–E18.5 which are closest to our experimental material considered below at E18.5 and P0 (DiI experiments). The mouse amygdalar pallial complex lies at the lateroventral caudal telencephalic region, where the lateral ventricle ends (Jacobowitz and Abbot 1997; Paxinos et al.^1994^; Puelles et al. 2000; Medina et al. 2004). This final region of the lateral ventricle separates the rostral pallial amygdala from the neighbouring caudal ends of the retrocommissural hippocampus, the entorhinal cortex, and the olfactory cortex, including the transitional amygdalopiriform area (following our recent analysis in Puelles et al. 2019, we interpreted the latter as para-amygdalar cortex intercalated between piriform and entorhinal cortex, that is, not as an amygdalar component).

**Fig. 1:**
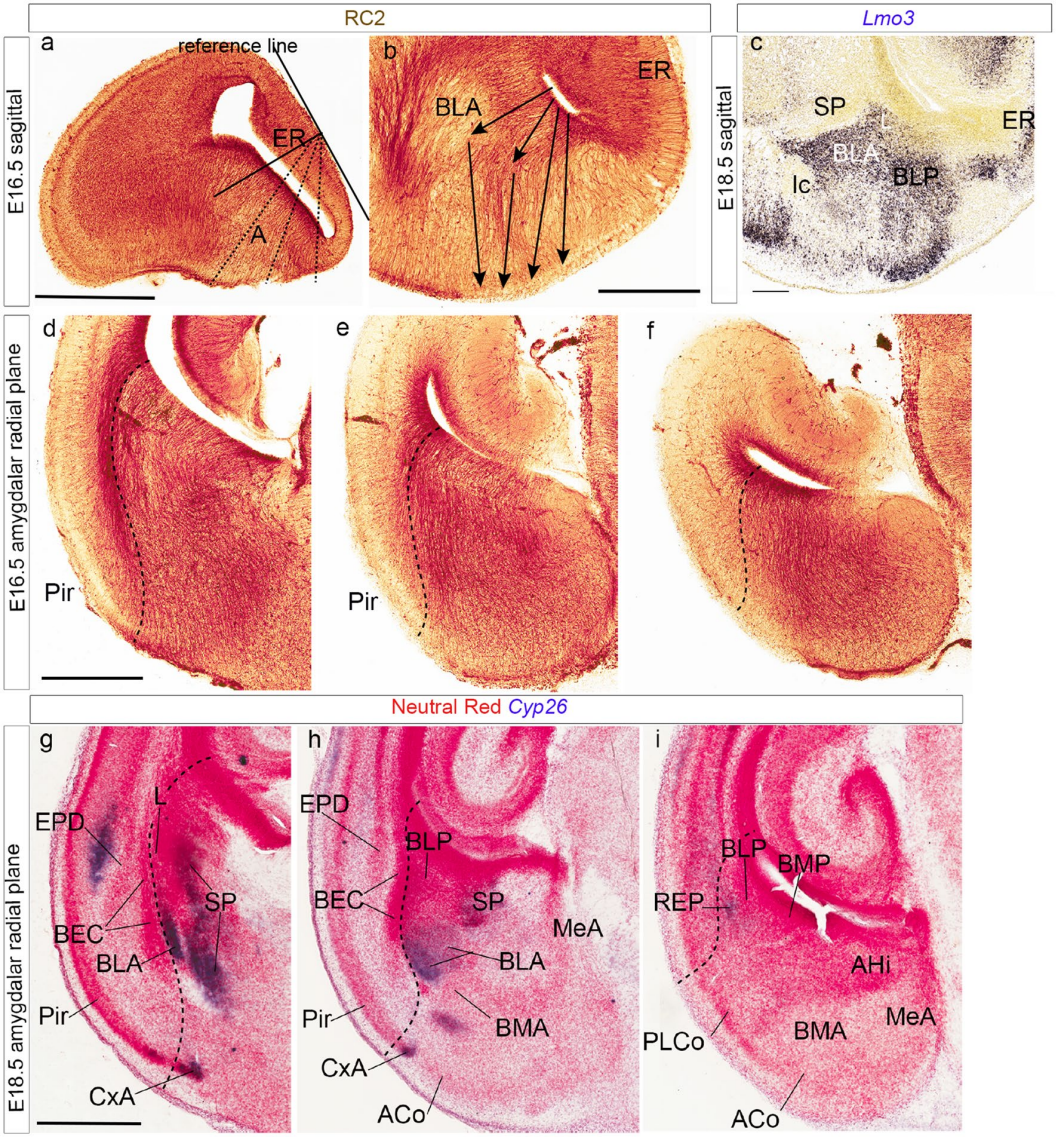
Amygdalar RC2-labelled radial glial organization and definition of a new amygdalar radial section plane. **a**,** b** Sagittal sections through embryonic mouse telencephalon immunostained with RC2 antibody at stage E16.5; a line tangent to the entorhinal cortex was drawn in **a** to serve as a reference for the obliquity of amygdalar glial processes and section planes (dotted lines). Arrows in **b** indicate gradual changes in the inclination of the observed glial processes, comparing with the ventricular and pial amygdalar surfaces. Note particularly that the dorsalmost glia associated to the L and BLA nuclei show a sharp knee-like ventral bend at the boundary of the subpallium. **c** sagittal section hybridized for *Lmo3* at E18.5, from the Allen Developing Mouse Brain Atlas. This gene marker labels the BLA nucleus and illustrates independently that the glial ventral knee-bend corresponds to the rostralmost tip of the nucleus. **d**–**f** Three sections obtained in the ‘amygdalar radial plane’ at three anteroposterior levels through the mouse telencephalon (from rostral to caudal), immunostained with RC2 at stage E16.5. The stained radial glial processes can be followed uniformly from the ventricle to the pial surface. The dash lines mark the boundary between pallial amygdala and neighboring olfactory cortex (i.e., ventral pallium; a dense packet of ventropallial radial glial processes is seen particularly in **d**). **g**–**i** Three sections obtained in the ‘amygdalar radial plane’ through rostral to caudal amygdalar levels, hybridized with *Cyp26* (blue reaction), and counter-stained with neutral red at stage E18.5. The dash lines mark the boundary between pallial amygdala and neighboring olfactory cortex. Note selective *Cyp26* signal at the superficial CxA (**g**, **h**) and the BLA (**g**–**i**). Note also independent expression in portions of neighboring subpallium (SP). Scale bars represent 600 µm (**a**,** c**), 800 µm (**b**) 400 µm (**d**–**f**) and 500 µm (**g**–**i**)

Sections through the pallial amygdala in any plane show the caudal end of the lateral ventricle as a flattened cavity closed medially by thin chorioidal tissue (the end of the chorioidal fissure; Figs. 1, 4, 5, 7). As observed in sagittal sections, the radial glial processes emanating from the ventricular zone in the amygdalar area follow more or less curved courses to reach the ventral pial surface, which lies roughly at a right angle relative to the ventricular plane. If we judge relative to a reference plane tangent to the flat entorhinal cortex, the ventriculo-pial glial processes course in an oblique plane gradually diminishing from 45° to 30°, or less, in rostrocaudal order (Fig. 1a, b). Indeed, the glial processes gradually become vertical (parallel to the reference plane) at the back of the amygdala, which extends topographically somewhat under the ventricle, close to its posterior boundary of the amygdalo-hippocampal area with the entorhinal and hippocampal regions (Fig. 1a). Contrarily, at the rostral end of the complex, where the pallial amygdala approaches its border with the striatal subpallium, the glial processes first proceed nearly horizontally rostralwards up to the rostral tip of the anterior basolateral nucleus (BLA; compare labelling with *Lmo3* ISH; Fig. 1b, c), passing under the striato-amygdalar area. Thereafter they form a knee-bend around the BLA, continuing ventralwards nearly vertically to reach the pial surface; these glial processes bent at the BLA originate at relatively more dorsal parts of the amygdalar ventricle (Fig. 1b); more ventral glial processes do not show this knee, and instead angle downwards in a smooth curve at about 45° inclination. We believe that the anomalous knee-shaped course suggests a local morphogenetic deformation of the radial dimension, probably caused by an unique mode of development of the massive intermediate BLA mass. Obviously, we must conclude from these changing angles that no single section plane can be optimal for all parts of the pallial amygdala. We accordingly decided to study the radial histogenetic units of the mouse amygdala in compromise section planes varying between 45° and 30° relative to the cited plane of reference, i.e., some brains were cut at approximately 45 degrees and others at 30°; given there always is a small error in judging the angle, in practice our ‘radial amygdalar’ sections oscillated between these extremes (Fig. 1a). The 45° angle is better for the rostral half of the amygdala, whereas the 30° angle resolves better the caudal half. We called all these planes oblique to standard coronal sections the ‘amygdalar radial plane’ (example in Fig. 1d–i), and used them for various purposes in the present study. These Figures illustrate in glia-immunoreacted (E16.5; Fig. 1d–f) and *Cyp26* ISH (E18.5; Fig. 1g–i) material how we could trace the radial boundary that separates the pallial amygdala from the olfactory ventral pallium all the way from the lateral ventricle to the pial surface (Pir versus CxA). Glial stains readily show a dense packet of glial processes which courses radially just outside of the amygdala (best visible at rostral and intermediate levels; Fig. 1d, e); this limiting packet of radial glia, which contains interstitially the bed nucleus of the external capsule (BEC; Fig. 1g, h; Puelles 2014; Puelles et al. 2016a, b) disappears (or bends around the caudal pole of the amygdala) caudal to the end of the olfactory cortex (Fig. 1f, i).

### Adult AZIN2-LacZ expression

Pallial amygdalar AZIN2-LacZ expression counterstained with either parvalbumin (PV), tyrosine hydroxylase (TH) or calretinin (CR) immunoreaction is a novel labelling approach which we found very useful to understand the pallial amygdala. This recently emerged neural marker is related to polyamine metabolism, the AZIN2 protein (antizyme inhibitor 2) being a non-ubiquitous non-functional analog of the ornithine-decarboxylase protein which counteracts the inhibitory activity on this enzyme of the three known antizymes AZ1-3 (López-Garcia et al. 2013). There is also an ubiquitous AZIN1 protein, whereas AZIN2 is present postnatally in a selective and highly reproducible pattern in diverse neuronal populations, including some amygdalar ones (Martinez-de-la-Torre et al. 2018; Puelles et al. 2019). The AZIN2 knockout has no obvious brain phenotype, probably due to functional redundancy with AZIN1.

**Fig. 2:**
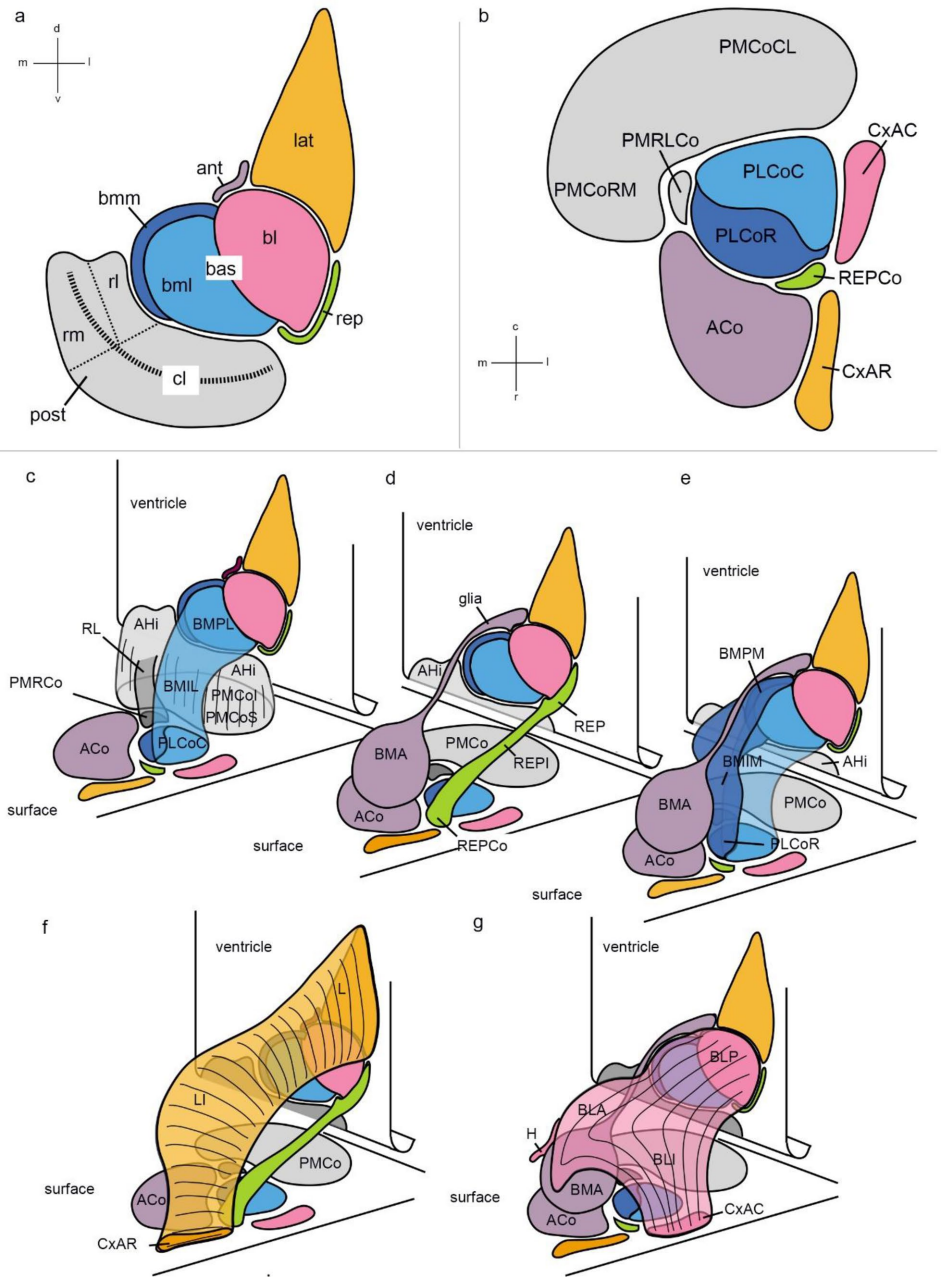
3D representation of pallial amygdalar radial units. **a** Flat representation *at the ventricular surface* of the five radial units (ant, lat, bas, post, rep). Note bl, bml and bmm are subdivisions of the *basal* unit. Similarly, cl, rl and rm are subdivisions of the *posterior* unit. The striped curved line across the *posterior* unit represents the approximate projection of the fundus of the lateral ventricle; the portion underneath this line approaches caudally the hippocampal cortex. **b** Flat projection upon the brain surface of the superficial amygdalar structures related to the radial units (same color code as in **a**). **c**–**g** 3D schemata representing various amygdalar radial units in the context of the ventricular and pial surface maps sketched in **a**, **b**, which are copied in perspective; note only part of the ventricular surface of the *posterior* unit is seen, because the rest bends under the symbolic end of the lateral ventricle. **c** 3D-representation of the *basomedio-lateral* subunit (light blue) and *posterior* unit (light grey), **d** *rep* (green) and *anterior* (purple) radial units, **e** *anterior* unit (purple) and the bml (light blue) and bmm (dark blue) subunits. **f** *lateral* radial unit (light orange) and *rep* (green) unit. **g** *basolateral* subunit (pink) and *anterior* unit (purple)

**Fig. 3:**
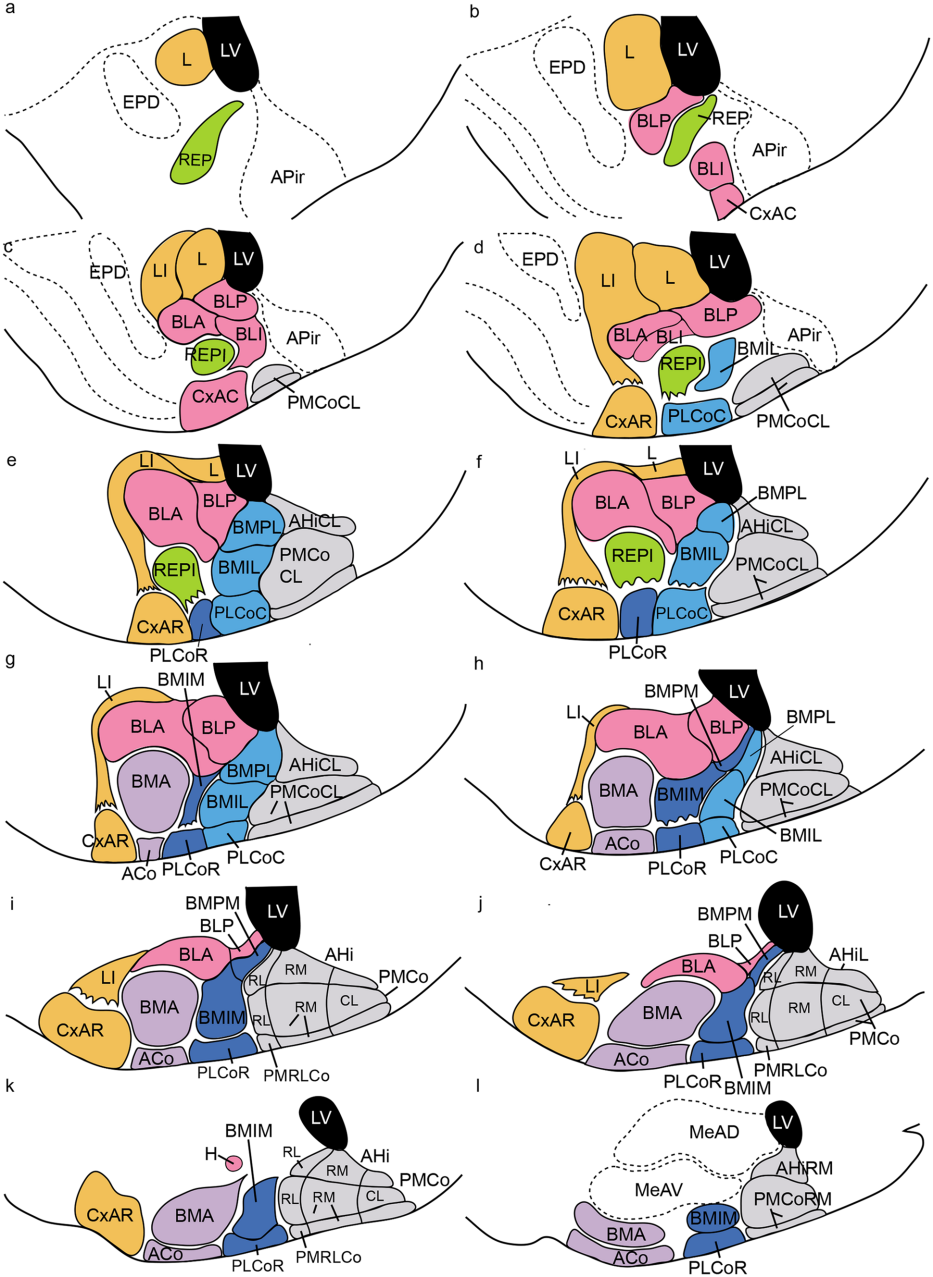
Color-coded graphic schematics of postulated amygdalar radial units mapped upon atlas sagittal planes. The tracings were reproduced with suitable modifications from consecutive schemata published in the mouse adult brain atlas of Paxinos and Franklin (2013; Atlas Figs. 131–118). The color codes are identical with those used in Fig. 2. This figure should be compared with microphotographic images in Fig. 7

**Fig. 4:**
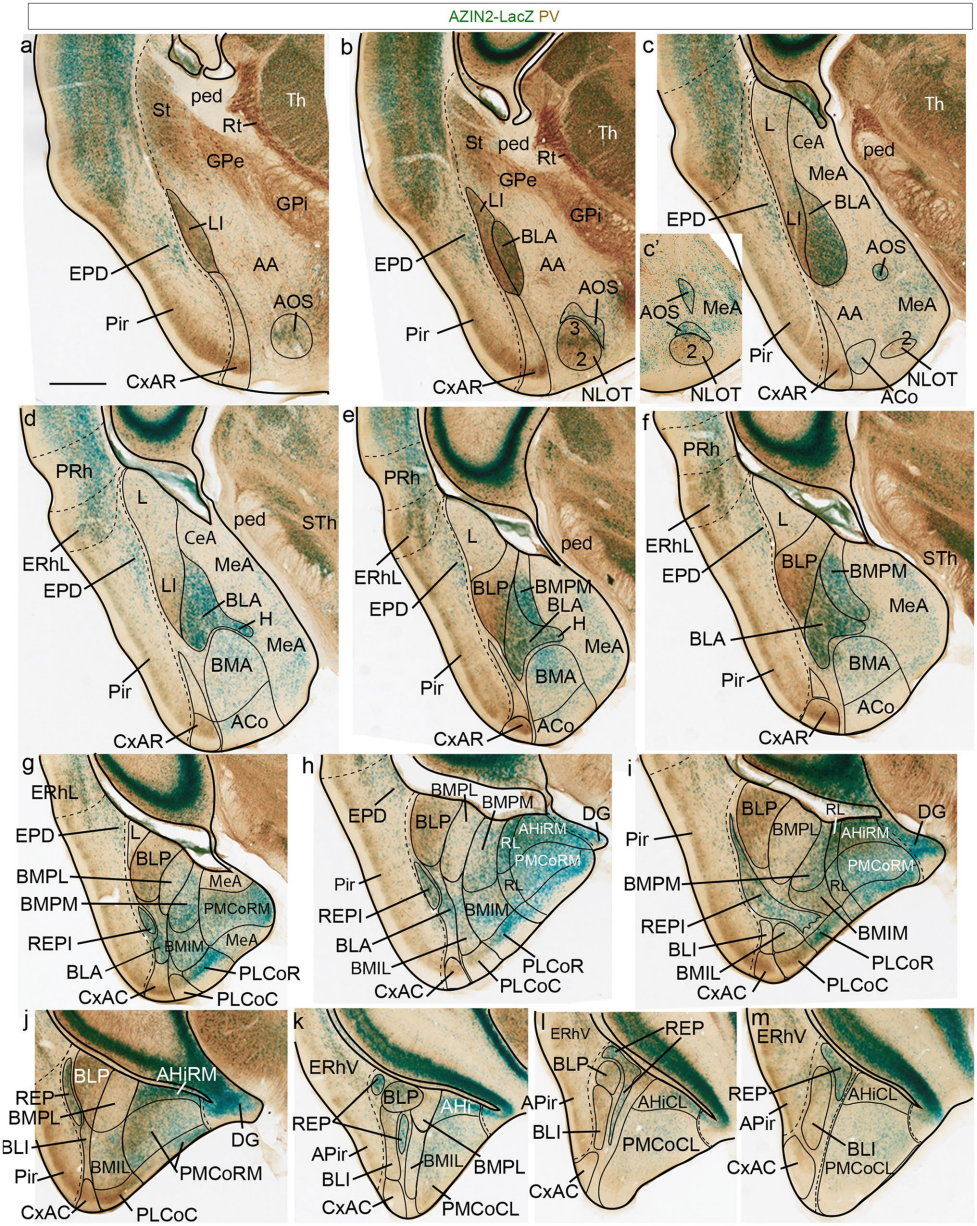
AZIN2-LacZ expression in adult amygdala, in amygdalar radial plane sections. AZIN2-LacZ expression (green signal) counterstained with PV (brown reaction) in amygdalar radial planes (at 30° to the entorhinal reference plane) in adult mouse, ordered from rostral to caudal levels. The limit between pallial amygdala and various cortical areas is indicated with a black dashed line. The rostralmost sections (**a**,** b**) show the boundary between pallial amygdala components and various caudal ganglionic eminence (subpallium) elements (St, GPe, GPi, AA). After **c** the pallial amygdala reaches the ventricular surface laterally to the subpallial central and medial amygdala. The postulated radial units are separated by thin black lines. Note the PV-positive neuropile of the LI and BLP nuclei (**a**–**j**). The AOS stream and NLOT are seen in **a**–**c**, before the former meets the BLA horn (H; **d**, **e**). Differentially labelled caudal and rostral parts of the PLCo are seen in **g**–**i**. BMPL and BMPM are also distinguished side by side in **g**–**i**. Scale bar represents 900 µm

**Fig. 5:**
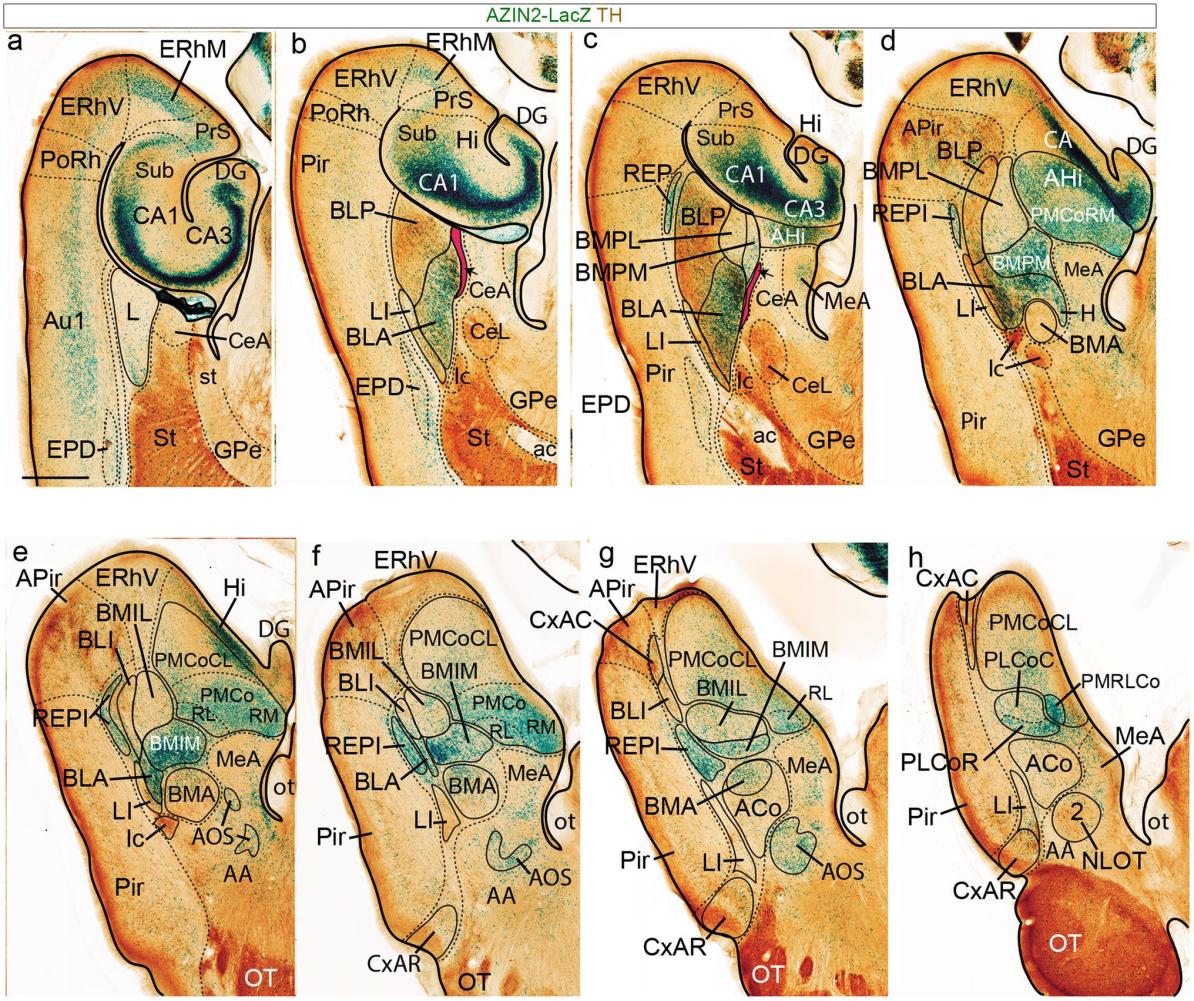
AZIN2-LacZ expression in adult amygdala, horizontal sections**.** Horizontally sectioned AZIN2-LacZ material (green signal) through the adult amygdalar region was counterstained with TH (brown reaction; dorsal to ventral order). The lateral limit of the amygdala, various cortical limits and some subpallial limits are indicated by black dash lines. Sections **a**–**c** show the amygdalo-hippocampal end of the lateral ventricle. The remaining levels show structure found *under* the ventricle. At levels **b**, **c** we delineated in red how we visualize the glial packet corresponding to the *anterior* radial unit as it approaches the ventricle next to BLP (black arrow). Sections **e**–**h** are useful for visualizing the differentially AZIN2-labelled BMIL and BMIM nuclei. See also the novel superficial PMRLCo element in **h**, compared to the two parts of PLCo proper. Superficial REPCo is seen (unlabelled) in **g**. Scale bar represents 900 µm

**Fig. 6:**
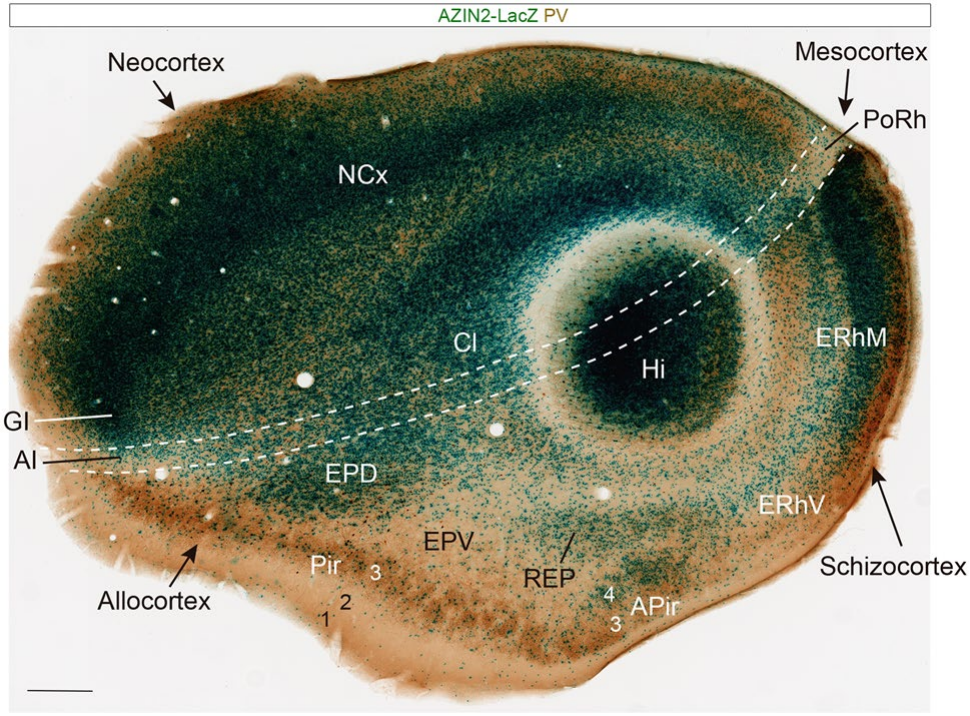
Sagittal section through AZIN2-LacZ-stained and PV-counterstained adult brain which barely grazes the lateralmost part of the amygdala. This sagittal overview section shows schematically the position of the pallial amygdala relative to the general pallial division. There is a large central island of neocortex (NCx), surrounded by a ring of mesocortex (symbolized by white dash-lines) and a broader outer ring of allocortex. The latter shows at this section level three distinct parts: the olfactory allocortex (Pir), the variant entorhinal allocortex, or schizocortex (ERhV/ERhM) and the largely invaginated hippocampal allocortex (Hi; relevant transitions are better seen in more medial sections). Deep to the insular mesocortex (AI) there appears the claustrum (Cl). Deep to the Pir there are the dorsal and ventral endopiriform nuclei (EPD, EPV), both of which approach, but do not overlap with, the independent pallial amygdala. The Figure shows that the amygdalo-piriform area (APir) lies selectively at the ventral transition of Pir into ERhV (APir does not reach dorsally the border with mesocortex). The only visible part of the pallial amygdala is the REP nucleus, found next to the APir (its thicker apparent rostral end corresponds to the classic BLV nucleus). Scale bar represents 2 mm

**Fig. 7:**
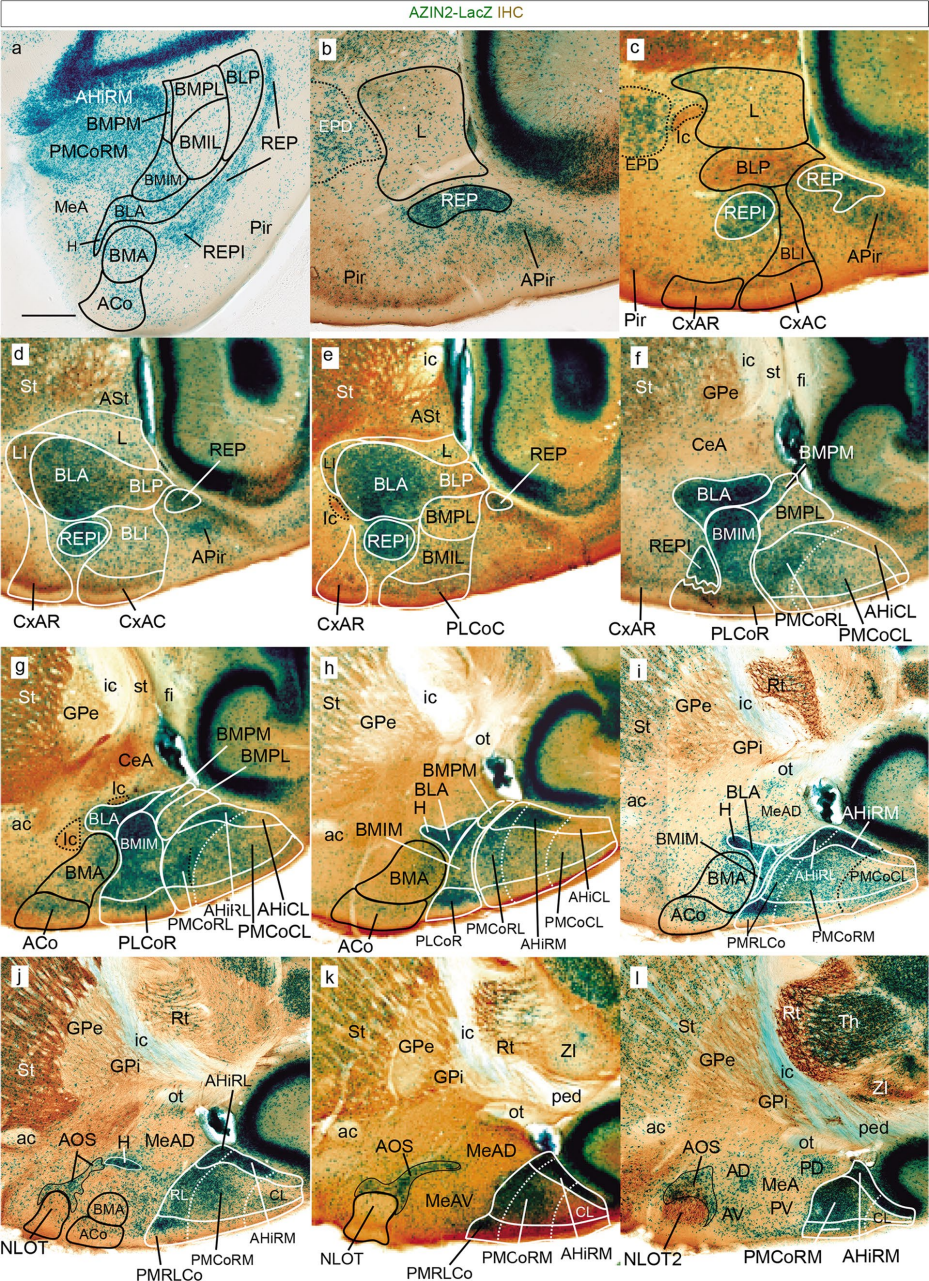
Sagittal sections with AZIN2-LacZ expression in adult amygdala. The panel **a** shows an amygdalar radial section illustrating optimal visualization of the AZIN2-LacZ stained REP nucleus mentioned in Fig. 4 (no counterstain), seen here extending homogeneously from the ventricle lateral to BLP into the classic BLV cell aggregate (renamed here REPI) next to BMA and BLA. This continuity had not been observed previously. The following panels (**b**–**l**) are all sagittal sections (lateral to medial order, rostral to the left; dorsal up) showing AZIN2-LacZ expression counterstained with either PV, TH or CR in the pallial amygdalar region of adult mouse (indicated at lower right corner). The REP/REPI is here visualized at sagittal section levels deeper than Fig. 4 (panels **b**–**f**), extending beyond the large BLA mass into the rostralmost part of the PLCo complex (**f**; alternatively understood as a separate REPCo nucleus). Medially next to this outermost unit we see the L-LI-CxAR radial complex (**b**–**e**) and the BLP-BLI/BLA-CxAC radial complex (**c**, **d**). Note the horn of the BLA (H; **h**–**j**), in continuity with the AOS formation reaching the NLOT (AOS; NLOT; **j**–**l**).The rostral and caudal parts of the PLCo (**e**–**h**) appear connected in depth via the BMIM and BMIL intermediate nuclei with the periventricular parts of the classic BMP (BMPM, BMPL; **e**–**i**). The BMA-ACo complex shows no continuity with any deeper nuclei (**g**–**j**). Finally, the *posterior* radial unit reveals its three strata and molecular CL, RM and RL subdivisions (**f**–**l**). Scale bar represents (**a**) (**b**–**c**) (**d**–**m**). Scale bar represents 900 µm

Here we studied in detail AZIN2 distribution in the amygdalar neighborhood, using heterozygotes from the available AZIN2-LacZ mouse line (details in Martinez-de-la-Torre et al. 2018). To make the description easier, we will first summarize the radial model that was finally obtained, which will guide the reader through our subsequent description (see Figs. 2, 3). In general, we aimed at minimizing name changes. We will illustrate first adult AZIN2-LacZ brain sections cut obliquely at 30 degrees relative to the plane tangent to the entorhinal cortex and counterstained with parvalbumin immunoreaction (AZIN2-LacZ/PV; Fig. 4). This material is compared with a TH-counterstained AZIN2-LacZ horizontal section series (AZIN2-LacZ/TH; Fig. 5) and a PV, TH or CR-counterstained sagittal section series (AZIN2-LacZ/PV/TH/CR; Figs. 6, 7). The three series show consistent differential staining characteristics inside some of the classic amygdalar nuclei, which we think aid understanding their radial relationships, as well as their mutual planar positions.

### Radial model

Our present results are consistent with a model in which the pallial amygdala is formed by 5 fundamental radial histogenetic units identified as *lateral, basal, anterior,* *posterior* and *retroendopiriform* domains (Figs. 2, 3, 4, 5, 6, 7). The basal and posterior units are each further subdivided molecularly into 3 subunits, so that in fact we have 9 different radial structures to consider. There is a novel *retroendopiriform* radial unit (classically not contemplated in amygdalar schemata) corresponding to the *retroendopiriform nucleus*, which apparently includes the classic ventral basolateral nucleus (BLV, now renamed REPI), and has a distinct amygdalar superficial end (REPCo). We favour the possibility that it may be a true radial unit of the pallial amygdala, parallel to the other units, rather than a para-amygdalar structure topographically related to amygdalo-piriform cortex, as is habitually conceived.

The *lateral* radial unit lies relatively dorsal and somewhat more rostral to the rest (next to the striatal subpallium). It is mainly represented by its classic periventricular element, the *lateral* nucleus (L), and ends at the superficial *rostral cortex-amygdala* transitional formation (CxAR; classic CxA). These ends are interconnected radially by what we call the *intermediate lateral* nucleus (LI; this term is new, but refers explicitly to the lateralmost part of the old BLA, already previously distinguished in the literature).

The *basal* radial unit is larger than the lateral one. It encompasses periventricularly the sum of the classic *posterior basolateral nucleus*, BLP, and the *posterior basomedial nucleus*, BMP. It appears subdivided molecularly into *three* parts, to which we gave names inspired in their periventricular element, i.e., the *basolateral*, *basomedio-lateral* and *basomedio-medial* subunits (the implied division of the classic BMP nucleus in two parts is new, based on AZIN2-LacZ data).

The *basolateral subunit* comprises the periventricular BLP nucleus and, in this case, two intermediate masses, the new BLA nucleus (including a substantial part of the conventional BLA, minus the LI mentioned above) plus a novel *intermediate basolateral nucleus* (BLI), complementary to the BLA*,* which ends superficially at the *caudal cortex-amygdala* transitional formation (CxAC).

The accompanying *basomedio-lateral subunit* includes the ventrolateral part of the classic BMP (BMPL), and a corresponding novel *basomedial-lateral intermediate* cell mass (BMIL), which ends superficially at the caudal part of the *posterolateral cortical nucleus* (PLCo).

The *basomedio-medial subunit* is clearly distinguished molecularly from its companion (see below). It includes periventricularly the dorsomedial part of the classic BMP (BMPM), has at intermediate levels a novel *basomedio-medial intermediate* cell mass (BMIM), and ends superficially at the rostral part of the PLCo. Importantly, our model holds that the classic BMP is *not related radially* with the classic BMA nucleus, which belongs rather to the *anterior* radial unit (clearcut molecular evidence supporting this point is shown below).

The *anterior* radial unit is singular, because all its derivatives accumulate during development at the adult intermediate and superficial strata, formed, respectively, by the conventional *anterior basomedial nucleus* (BMA) and the anterior cortical nucleus (ACo); some other derivatives from this unit apparently migrate tangentially into neighbouring subpallial areas, including anterior amygdala and medial amygdala subregions. This *anterior* unit, therefore, lacks a recognizable cell population at periventricular level, being represented there exclusively by a cryptic palisade of radial glia cells. This *anterior* unit essentially separates the *lateral* and *basal* units from the pallio-subpallial boundary (Fig. 2a, d).

The *posterior* or *amygdalo-hippocampal* radial unit extends caudalward under the ventricle until it meets the hippocampus, to whose area of molecular influence it may belong (Abellán et al. 2014). This large unit is represented periventricularly by the classic amygdalo-hippocampal area (AHi), whereas its intermediate and superficial levels are represented, as a whole, by the conventional posteromedial cortical nucleus (PMCo); the latter can be subdivided into a superficial corticoid stratum –the true superficial element- and a more voluminous intermediate inner stratum. Apart of these strata, various genoarchitectonic markers (see Table 4 and Suppl. Figs. 2, 3) reveal a subdivision of this *posterior* unit into subtly different rostromedial, rostrolateral and caudolateral portions, a notion already partly found in the literature (AHi*RM*/PMCo*RM*, AHi*RL*/PMCo*RL*, AHi*CL*/PMCo*CL*; see Discussion). The PMCoRL portion separates from the rest of the posterior unit as it approaches the brain surface, and ends either at the medial border of the PLCo, or as a small distinct separate corticoid patch along that medial border (Fig. 3). The *retroendopiriform* unit (*rep*) presents periventricularly the conventional REP nucleus, found caudally lateral to the BLP (and deep to the APir cortical area), as identified in recent rodent brain atlases (note some literature wrongly identifies it as ‘ventral endopiriform nucleus’). The *rep* appears selectively labelled by several gene markers, as well as in our AZIN2-LacZ material; the latter clearly shows that this formation extends superficialward in the oblique radial amygdalar plane until it fuses with the conventional ventral basolateral nucleus (here renamed intermediate REP nucleus; REPI; Suppl Fig.1a), which represents the intermediate stratum of this unit (coronal sections falsely tend to show these elements as discontinuous). The superficial end of the unit occurs either at the rostral border of the PLCo, or in a small separate corticoid patch just rostral to PLCo (named here REPCo; Figs. 2d, 3; Suppl. Fig. 2).

As regards mutual relationships (Fig. 2), the *lateral* unit contacts only the *basolateral* and *anterior* subunits. The latter is the unit which contacts rostrally the subpallium, as noted particularly at the intermediate and superficial levels (at periventricular levels the absence of a visible *anterior* component produces the impression that the L nucleus contacts the striatum, though we hold that they are separated by the glial palisade of the *anterior* unit). The *basolateral* unit is deformed radially, forming the cited rostrally prominent knee at intermediate levels (BLA). In addition, some basolateral derivatives are found outside of the strict radial limits of this unit. For instance, a sizeable non-radial medial extension of BLA aggregates as the ‘BLA cap’ above the BMA nucleus. This non-radial medial BLA extension finishes in what we call the ‘BLA horn’, a rostrally pointing curved horn-shaped process which protrudes medially into the subpallial amygdala neighbourhood; in the Discussion we argue that this non-radial peculiarity possibly implies a tangential migration of a group of BLA cells around the BMA mass. Other data commented below further relate *indirectly* the BLA horn to the migration route of the NLOT nucleus. The *basolateral* unit contacts medially the BMPL and BMPM subunits (mainly the former). Both the *anterior* and *posterior* radial units contact the medial amygdala, which is considered subpallial in the field (e.g., Swanson and Petrovich 1998).

These radial units are represented tridimensionally and color-coded as an artist drawing in Fig. 2, and we also show for tri-dimensional precision an identically color-coded perspective of this model in Fig. 3, based on a suitably modified interpretation (after inclusion of our novel subnuclei) of mouse adult brain sagittal atlas plates 131–118 from Paxinos and Franklin (2013); Fig. 3 can be compared with our AZIN2-LacZ sagittal section series in Fig. 7. Our model units are also mapped through a hemisphere sectioned in topologically transversal sections to the secondary prosencephalon (i.e., parallel to the peduncular hypothalamus) in Suppl. Fig. 2.

### AZIN2-LacZ/PV, AZIN2-LacZ/TH, and AZIN2-LacZ/CR adult series

We proceed now to describe examples of our AZIN2-LacZ material across a radially sectioned AZIN2-LacZ/PV series (Fig. 4), a horizontally sectioned AZIN2-LacZ/TH series (Fig. 5), and a sagittally sectioned AZIN2-LacZ/PV/TH/CR series (Figs. 6, 7). Close section series are needed in every case, because otherwise, we only glimpse here and there at some relationships (much literature only shows such glimpses), but cannot trace confidently units from the ventricle to the pial surface across the whole field of interest. The radially sectioned AZIN2-LacZ series (Fig. 4) leads in the recognition of radial connectivity (what continues into what). Fine adjustment of the amygdalar radial section plane aids the optimal visualization of some details (we selected for publication those shown in Fig. 4, and we included in other Figures some extra images to illustrate the appearance in a slightly different plane). The horizontal and sagittal AZIN2-LacZ series (Figs. 5, 6, 7) show complementary corroborating evidence for our description. They can be seen as independent tests of the consistency of our interpretation, jointly with Fig. 3 and Suppl. Fig. 2. Additional helpful detail is provided by the counterstaining applied to the AZIN2-LacZ material, namely either PV, TH or CR immunoreaction. We also considered, and made our interpretations consistent with, the AChE-reacted plates shown in several rodent brain atlases.

Figure 4a, b of the radial series show the rostralmost appearance of the pallial amygdala, cut at the BLA-related glial knee mentioned above. This involves the rostrally protruding intermediate components of both the *lateral* and *basolateral* radial histogenetic units (LI; BLA). The thin *lateral intermediate nucleus* –LI- is represented by a PV-positive band with scarce AZIN2-LacZ-positive cells which contours laterally the conventional BLA nucleus, and fuses backwards with the L nucleus (LI; Figs. 4a–d, 5b–h, 7d, e). The periventricular L neuropil is instead PV-negative, much broader than LI, and displays a sparse population of small AZIN2-positive cells (L; Figs. 4c–g, 5a, 7b–e). The subdivisions of L identified in most rodent atlases and other literature were not distinguished in our AZIN2-LacZ material. The LI points ventrally pialwards into the rostral part of the cortex–amygdala transition zone (CxAR), after crossing a cell-poor stratum containing only some dispersed small AZIN2-LacZ-positive cells (compare Fig. 4g, Fig. 5 and 7d, e). The CxAR shows some background PV and TH immunoreaction, which in both cases appears slightly stronger than within the adjacent piriform cortex (LI; CxAR; Pir; Figs. 4a–f, 5b–h, 7c–f); distinct AChE activity also appears at the CxAR (Paxinos and Franklin 2013).

Immediately medial to LI there appears the rostral tip of the BLA knee as a lentiform PV-positive neuropil which contains a significant population of magnocellular AZIN2-LacZ-positive neurons. We think that the conventional BLA nucleus essentially occupies the *deep intermediate* bent portion of the *basolateral* radial subunit. More caudally the lateral end of the BLA extends into a more superficial, previously unrecognized *outer intermediate* mass, which we identify as the *intermediate BL* nucleus or BLI (BLA; BLI; Figs. 4b–m, 5b–g, 7a, c–e, Supp. Fig. 1n). The BLI nucleus points ventralwards into the underlying caudal part of the CxA, where the *basolateral* radial unit ends superficially (BLI; CxAC; Figs. 2, 3, 4g-m, 5e–h, 7c, d). CxAR and CxAC border laterally on the piriform cortex of the ventral pallium. Medialwards they both relate to the lateral edge of the PLCo superficial formation (associated to the 2 *basomedial* radial subunits). In opposite radial direction, towards the ventricle, the BLA mass is clearly continuous with the large periventricular BLP nucleus, which typically has a PV-, AChE- and TH-positive neuropil, with dispersed AZIN2-LacZ-positive cells (BLP; Figs. 4e–l, 5b–d, 7a, c–e; for the AChE, see Paxinos and Franklin 2013 and other rodent atlases). Next to the ventricle the BLP lies first ventromedial to L (with a clear-cut cytoarchitectonic limit), and then gradually substitutes it caudally, thus defining the caudomedial border of the *lateral* radial unit.

**Fig. 8:**
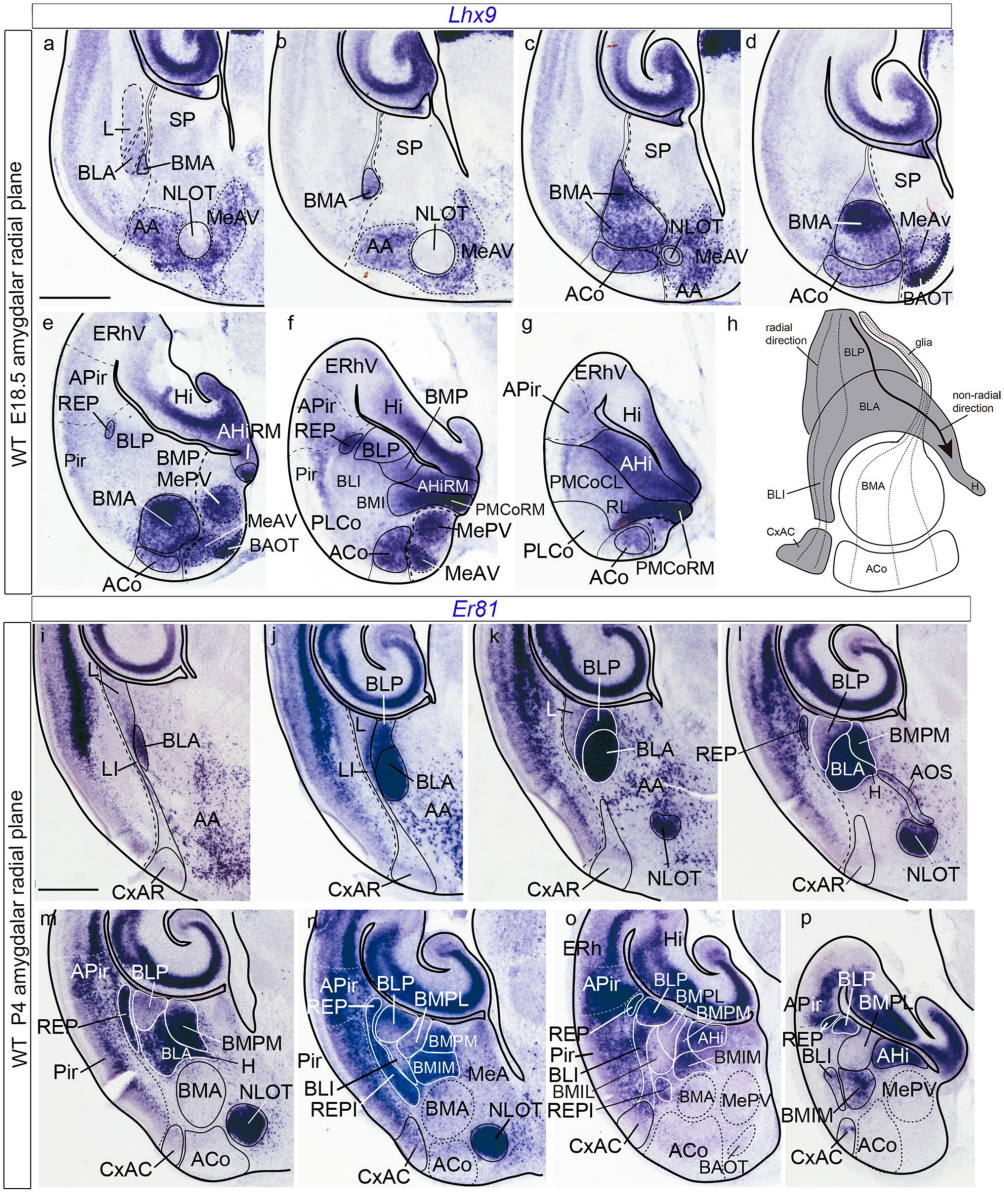
*Lhx9* expression and *Er81* expression in the perinatal amygdala. **a**–**g** *Lhx9* expression in a series of sections cut in the amygdalar radial plane through the mouse amygdala at E18.5, ordered from rostral to caudal. **a**, **b** Rostral levels show a small cap portion of the BMA, labelled adjacent to the BLA, jointly with superficially migrated positive cells in AA and MeAV surrounding the unlabelled NLOT nucleus. **c**, **d** These section levels show the rostral part of BMA, with associated ACo nucleus, and superficially migrated cells in AA and MeAV, as well as the positive BAOT nucleus (this may be independent of the *anterior* unit; see Suppl. Table 1). **e**–**g** caudal levels through BMA and ACo, BAOT, and migrated cells in MeAV, MePV. Independently, there is labelling at the *rep* (**e**, **f**), AHiRM and PMCoRM (**f**,** g**). **h** Schematic drawing of an amygdalar radial section through the *anterior* and *basolateral* radial units to show the disposition of radial glial processes: in the *basolateral* unit they pass from the superficial CxAC and intermediate BLI into the lateral half of the BLA, before reaching the periventricular BLP (the black arrow indicates apparent tangential displacement in medial direction of a contingent of BLA cells, to end at the BLA horn [H]). In the *anterior* unit the glial fibers pass from the superficial ACo through the intermediate BMA into a thin packet of radial glia processes which course along the medial side of the BLP into the ventricle (i.e., not entering the BMP). **i**–**p** *Er81* gene expression in amygdalar radial sections at P4. The black dash-lines mark the limit between the pallial amygdala and neighboring cortical areas. Note positive AOS cells in **l** and REP and REPI in **l**–**p**. Scale bars represent 600 µm (**a**–**g**) and 700 µm (**i**–**p**)

The large cells of the intermediate BLA mass characteristically also build a cap-shaped medial extension above the BMA nucleus (this is also clearly visible in conventional coronal sections). This cap population seems to depart medialwards from the straight radial route through BLP/BLA into BLI and CxAC (Figs. 4d–m, 7c, d, 8h). The BLA cap over the BMA ends characteristically in a medially oriented horn-like AZIN2-LacZ-positive process (tagged here as H) which protrudes past the BMA into the adjacent amygdalar subpallial territory (MeA), pointing slightly rostralward (H; BLA; BMA; Figs. 4d, e, 5d, 7h–j). According to various rodent brain atlas data, H is selectively AChE-positive, like the BLA cap and the BLA and BLP in general; this result argues for its derivation from the *basolateral* radial unit. Interestingly, AZIN2-LacZ-expression also extends rostralward beyond the medial BLA horn via a delicate, somewhat disperse, rostrally-directed linear stream of AZIN2-LacZ-positive (but AChE-negative) cells, which eventually reaches the NLOT nucleus (n. of the lateral olfactory tract). Similar cells distribute there mostly within its layer 3, and form also a tenuous shell around the whole NLOT, without entering its layers 2 or 1. We named this apparently novel amygdalar entity the *amygdalo-olfactory stream* (AOS; Figs. 4a–cʹ, 5e–g, 7j–l). As mentioned, the more compact medial BLA horn (H) is clearly AChE positive (e.g., Paxinos and Franklin 2013; Plate 138, left side, and Plates 121, 123), but not so the less compact AOS continuation into the NLOT, which, therefore, is a distinct cell population, irrespective of sharing the AZIN2-LacZ signal. Importantly, the AOS component was later corroborated selectively with some other markers, such as *Er81* (AOS; Fig. 8).

Medially to the BLA/BLP transition there soon appear in the radial series periventricular and intermediate portions of the classic BMP nucleus. This has always been assumed to be a relatively homogeneous population held to be continuous rostrally with the BMA. In contrast, our AZIN2-LacZ material shows a neat division of the classic periventricular BMP into distinct AZIN2-LacZ-positive and AZIN2-LacZ-negative portions (these were announced above as *BMPM* and *BMPL*, respectively; see Figs. 2, 3). Note the AZIN2-LacZ-positive BMPM is shaped periventricularly as a curved domain that first covers *dorsally* the BMPL, extending over it as a slender extension which contacts briefly the BPL, and then becomes restricted to the wider medial side of BMP, next to the AHi area; in contrast, the AZIN2-LacZ-negative BMPL has a roundish shape periventricularly (BMPM; BMPL; BLP; Figs. 4e–j, 5c, d, 7a, e–h; Suppl. Fig. 1q–s, v–x). Surprisingly, none of these two radial subunit streams are continuous with the intermediate BMA nucleus, from which they are separated by the medial cap-like extension of the BLA. These two *basomedial* subunits reach instead via newly recognized intermediate AZIN2-LacZ-positive *BMIM* and AZIN2-LacZ-negative *BMIL* masses separate likewise AZIN2-positive and AZIN2-LacZ-negative parts of the superficial PLCo formation (*PLCoR*; *PLCoC*). This new pattern is possibly best appreciated in horizontal sections (Fig. 5e–h; but see also Figs. 2c, e, 3).

BMPM can be seen in Figs. 4e–i, 5c, d, 7a, f–h, and the related intermediate mass BMIM appears in Figs. 4g–i, 5e–g, 7a, f–i. AZIN2-LacZ staining is equally strong in BLA and BMIM, which might suggest their continuity, but we noticed that TH, PV and AChE reactions are restricted to BLA and absent from BMIM. This dissociation of BLA and BMIM was also corroborated by other gene markers (e.g., Table 4). We further identified BMPL in Figs. 4g–k, 5c, d, 7a, e–g and its intermediate mass BMIL as AZIN2-LacZ-negative domains in Figs. 4h–k, 5e–g, 7a, e. There is accordingly an unexpected molecular bipartition of the classic BMP mass, coherently forming distinct parallel radial streams which bend radially pialwards (ventrally) into the PLCo. The latter corticoid area receives the BMIL in its AZIN2-LacZ-negative and PV-positive *caudal* part (PLCoC; Figs. 4g–j, 5h, 7e), while the BMIM reaches the AZIN2-LacZ-positive and PV-negative *rostral* counterpart (PLCoR; Figs. 4g–i, 5h, 7f–h). The medial border of what is conventionally identified as PLCo appears separately intensely positive for AZIN2-LacZ. We have found that it actually is not part of PLCo in terms of radial structure, since it represents the superficial *rostrolateral* subcomponent of the *posterior* radial unit (AHi/PMCo complex), which adopts an ambiguous position very close to PLCo (e.g., RL; PMRLCo; Figs. 3, 5e–h, 7i–k; Suppl. Fig. 2). Moreover, the rostral border patch of the PLCoR seems associated to the superficial end of the new REP radial unit (REPCo; Figs. 2c, e, 3, 7f; Suppl. Fig. 2).

The *anterior* radial unit contains in the adult mouse only the intermediate BMA nucleus and the superficial ACo area (Fig. 2d). These amygdalar elements are usually easily distinguished in our AZIN2-LacZ material, although they do not exhibit themselves much AZIN2-LacZ signal. The BMA characteristically is a rounded mass ending in a small conical tail which is clearly defined with the *Lhx9* marker (BMA; Fig. 8a–d). The BMA is capped by the BLA, lacks PV, AChE or TH signal, and contains a relatively uniform, moderately dense, small-celled AZIN2-LacZ-positive population (BMA; Figs. 4d–f, 5d–g, 7g–j). This mass clearly relates radially to the subjacent superficial stratum formed by the sparsely AZIN2-LacZ-positive ACo formation (ACo; Figs. 4c–f, 5g, h, 7g–j). The ACo expands significantly at the amygdalar surface, ending caudally directly rostral to the REPCo and PLCo (Figs. 5h, 7g–j). BMA and ACo are separated by a small cell-poor stratum with few AZIN2-LacZ-positive cells. Both BMA and ACo limit medially with the medial amygdala region, which is conventionally subdivided in dorsal and ventral parts (MeA; MeAD; MeAV; Figs. 4c–g, 5c–h, 7i–l). The superficial *bed nucleus of the accessory olfactory tract* (BAOT) present in this area deep to the end of the lateral olfactory tract is not distinguished at all in AZIN2-LacZ material, though it expresses strongly *Lhx9* (this combined pattern suggests a separate origin); the BAOT lies ventromedially, not far from the medial end of the ACo (BAOT; Fig. 8d, e). The superficial part of the *anterior* unit borders rostrally with the anterior amygdala area (AA), a subpallial territory, wherein the NLOT is located (AA; NLOT; Figs. 4a–c, 5e–h, 7j–l). In the Discussion we will argue that molecularly distinct neuronal populations in AA and MeAV/MePV may represent tangentially migrated elements of the *anterior* radial unit (see Fig. 8a–f, and “Discussion”).

We postulate on the basis of developmental observations on *Lhx9* expression (a selective marker of the BMA/ACo formation) that this *anterior* radial unit lacks a periventricular component, because between stages E14.5 and E18.5 all its derivatives apparently migrate radially into the intermediate BMA and superficial ACo/AA/MeAV/MePV sites. We will show below that the packet of radial glia that corresponds to the BMA and ACo nuclei extends in a flattened fan shape towards the ventricle first in lateral contact with the BLA (possibly also the BMPM), and later with the L nucleus. It meets the ependyma intercalated between the L and the pallio-subpallial boundary (small arrows point to this mapped glial packet labelled in red in Fig. 5b, c; it was not marked in Figs. 4 and 7). This is the topologic origin of the radially continuous early embryonic *Lhx9*-positive *anterior* radial unit primordium (e.g., as reported at E12.5; e.g., in Tole et al. 2005, interpreted as ‘ventral pallium’).

Caudomedial to the *basal* radial unit there appears the *posterior* radial unit, which is wholly PV- and TH-negative, and is represented by the AHi periventricular domain (continuous with the hippocampus beyond the caudal end of the ventricle) and the intermediate/superficial PMCo nucleus. Indeed, the PMCo combines the expected intermediate and superficial radial strata; the latter portion appears in some genoarchitectonic preparations as a distinct dense subpial corticoid layer, which accompanies the AHi throughout its areal extent (e.g., see *Trh* signal; Allen Mouse Brain Atlas; see also Suppl. Fig. 1z, aa). The periventricular AHi area starts medially and caudally to the BMPM and BMPL periventricular formations, and also lies caudal to the MeA. We think AHi is transitional with respect to the CA3-CA1 portion of the hippocampal cortex, as can be observed in several of the series we show. In AZIN2-LacZ material the extensive AHi plus PMCo complex divides into partly distinct rostromedial, rostrolateral and caudolateral subdomains, which we have separated by dash lines in the figures (AHiRM; AHiRL; AHiCL; and PMCoRM; PMCoRL [or PMRLCo]; PMCoCL; Figs. 4g–m, 5c–h, 7f–l; for clarity, in some cases these long tags are simplified to RL or CL).

The *rostromedial* subdivision is the first to appear in our radial section series, just caudally to the end of the MePD, where AHiRM and PMCoRM reach the brain surface at the medial aspect of the hemisphere just in front of the hippocampal tip. This part lies close to the ventral end of the dentate gyrus and also shows the final taenial insertion of the chorioidal tela of the hemispheric chorioidal fissure (beyond the earlier insertion at the MePD; see MeA; AHiRM; DG; Fig. 4g–i). The whole AHiRM/PMCoRM *post* complex subunit shows intense AZIN2-LacZ reaction (AHiRM; PMCoRM; Figs. 4g–j, 5d–f, 7h–l).

Laterally to this *posterior* RM subdomain, we detected a distinct *rostrolateral* *posterior* radial subunit occupying roughly the locus, where the *posterior* radial unit contacts the BMPM. This RL subunit shows a particularly high number of AZIN2-LacZ-positive cells in its periventricular and intermediate/superficial zones (AHiRL, PMCoRL; Figs. 4h, i, 5e–h, 7f–k), as well as a distinct molecular profile (see Suppl. Table 1). The AZIN2-LacZ reaction at the superficial RL component is particularly strong and lies quite close to the PLCo, sometimes causing the impression that it represents a medial end of PLCo; however, since the latter essentially relates to the *basomedial* radial subunits, we prefer to distinguish it as a separate and novel corticoid element, the PMCoRL (Figs. 5h, 7i–k; unlabelled in Fig. 4h; see also Fig. 2b and Suppl. Fig. 2n).

In contrast with the RM and RL subdivisions, AZIN2-LacZ expression diminishes significantly in the large *caudolateral* part of AHi/PMCo complex, particularly next to the ventral subiculum, its immediate caudal neighbour, where the AZIN2-LacZ signal is quite weak (AHiCL; PMCoCL; Figs. 2a, 4k–m, 5e–h, 7f–l). All of Ammon’s horn areas appear strongly AZIN2-LacZ positive, in contrast to the sparsely labelled subiculum (Sub; CA1, CA3; Fig. 5a–c).

We include in the amygdalar pallial domain a differentially labelled AZIN2-LacZ-positive cell population (also recognized by other genoarchitectonic markers; see below), which has been identified recently as the *retroendopiriform nucleus* (REP). This was mapped only close to the ependyma in the Paxinos and Franklin (2013) mouse brain atlas (visible adjacent to the BLP in their coronal Nissl-stained Plates 51–55). However, AZIN2-LacZ labelling detects this population not only periventricularly (REP; Figs. 4k–m, 5c, 6, 7c–e), but also as a continuous narrow radial prolongation lying deep to the APir cortical formation, which extends laterally past the BLP/BLA/BLI radial unit to a position just rostral to BLI. Here it apparently encompasses, fuses, or mixes with what is conventionally called the BLV nucleus, identified by us as *intermediate* REP nucleus (REPI; it seems inconvenient to mix this cell group with the *basolateral* radial unit). The REPI then deviates slightly ventromedially to meet what seems the rostralmost part of the PLCo. Given the essentially different topologic position of the *rep* formation with respect to the *basomedio-medial* radial unit ending rostrally at the PLCo, a point also underlined by its distinct molecular profile compared to the other radial units (Table 4), we are inclined to think that the superficial end of *rep* corresponds to an extra small corticoid formation, the REPCo, rather than it being a part of the PLCo proper (see REPCo; Suppl. Fig. 2n). The cited *rep* sequence can be followed in sagittal sections (e.g., REP; REPI; Fig. 7b–f), or in horizontal sections (REP; REPI; Fig. 5c–g). A particularly favourable amygdalar radial section plane shows practically the whole extent of *rep* from ventricle to brain surface (REP; REPI; Figs. 4g–m, 7a). Standard coronal sections only can show fragments of the REP radial unit; this probably explains that this continuity of REP with BLV/REPI and REPCo was not recognized previously. The ACo expands significantly at the amygdalar surface, ending caudally directly rostral to the PLCo (Figs. 5h, 7g–j).

### Other surrounding areas (APir, EPD, AA, MeA)

We found that AZIN2-LacZ material also was useful for characterizing the amygdalo-piriform area (APir), a formation classically ascribed to pallial amygdala, which according to recent results strictly lies outside the overall radial configuration of the pallial amygdala (Puelles et al. 2019). APir lies close to the REP and the *basolateral* part of the *basal* radial domain (APir; Fig. 5d–g; see also Fig. 6). Serial horizontal sections best illustrate that the APir lies intercalated between the caudal end of the periamygdalar piriform cortex (which extends rostral to APir) and the ventral part of the entorhinal cortex (extending caudally to APir; see APir; Pir; ERhV; Fig. 5a–g). The entorhinal area separates the APir from the ventral subiculum and the hippocampal allocortex, both of which contact the *posterior* radial unit of the pallial amygdala (see above). The ‘transitional amygdalo-piriform’ cortex area interestingly only appears at the topological *outer rim* of the caudalmost olfactory allocortex, close to the entorhinal cortex and to the pallial amygdala proper; the APir specialization is not detectable all the way across the local piriform allocortex to its limit with local perirhinal mesocortex (Fig. 6). It may be conjectured accordingly that amygdalar patterning effects perhaps cause the definition of this variant of the caudalmost piriform cortex, as a limited neighbourhood effect. Pir, APir, ERh and Hi cortical areas thus strictly *surround* on one side (lateral and caudally) the pallial amygdala, insofar as the 5 major amygdalar radial units described (counting *rep*) possess an independent ventricular and brain surface, the latter revealed by their respective corticoid superficial domains. The rostro-medial side of the pallial amygdala contacts the subpallium, including the caudal ganglionic eminence plus AA and MeA.

The molecular and pyramidal strata of APir stain in the present material similarly as those of the piriform cortex, showing scarce AZIN2-LacZ signal and some background PV immunoreaction, particularly in the cortical plate. However, APir uniquely shows a deep (layer 4) patch of higher AZIN2-LacZ expression next to REP (APir; Figs. 6, 7b–d), as well as irregular TH-immunoreactive and AChE-positive neuropil patches absent at the Pir (Fig. 5a–g; see AChE in Plate 131 of Paxinos and Franklin 2013).

Deep to the Pir there appears the dorsal endopiriform nucleus (EPD), which we interpret as a mass migrated tangentially from overlying, embryonic perirhinal claustral primordia (caudal EPD of Puelles 2014; Puelles et al. 2019); the EPD is characteristically found migrated ventrally to the insular and perirhinal cortex (lateral pallium; mesopallium) from where it supposedly originated. The caudal EPD contains a non-compact, relatively homogeneous population of weakly AZIN2-LacZ-positive neurons, whose position deep to periamygdalar Pir places it just lateral to the amygdalar capsule (i.e., caudal part of the external capsule), lying always adjacent to the amygdalar LI and L nuclei. In this topography it contrasts with the periventricular REP nucleus, which always lies just lateral to the BLP (EPD; Figs. 4a–h, 5a–c, 7b, c). It can accordingly be deduced that the REP lies caudal to the paraamygdalar EPD, rather than ventral to it, as is often assumed in some literature (i.e., note the REP is *not* a ventropallial EPV). The developmental origin of REP is still unclear and unexplored. However, the REP is clearly distinct molecularly from the EPD (i.e., is probably not lateropallial sensu Puelles 2014); notably, it does not express the characteristic lateropallial claustral *Nr4a2* marker (Puelles 2014), and it also differs molecularly from the EPV, a *Dbx1-LacZ*-positive ventropallial derivative which is indistinctive in our AZIN2-LacZ material (Fig. 6; Puelles et al. 2016a, b).

Our tracing of the pallio-subpallial boundary with a dash-line in various Figures is in general consistent with the standard notion that the AA and MeA territories can be ascribed to the amygdalar subpallium (though the NLOT and BAOT nuclei may represent non-subpallial populations migrated somehow into the AA; Remedios et al. 2007; García-Calero and Puelles, unpublished observations). The AA is poor in AZIN2-LacZ-labelled cells (AA; Figs. 4a–c, 5e–h), and it apparently receives many *Lhx9/Lhx2*-positive neurons from the *anterior* amygdalar radial unit (see below; Fig. 8). The MeA, which also apparently receives migrated *Lhx9*-positive neurons of the same origin (Fig. 8), displays a slightly higher density of dispersed AZIN2-LacZ neurons, which generally form a relatively dense aggregate in a stratum parallel to the brain surface; we did not notice significant differences in the AZIN2-LacZ pattern between the different parts of the MeA (MeA; MePD; MePV; MeAV; Figs. 4c–g, 5c–h, 7i–l).

### Ancillary gene pattern analysis at perinatal stages (*Lhx9, Er81*)

We mapped the expression pattern of *Lhx9* in E18.5 embryonic mouse and *Er81* in P4 mouse (material available in our collection). These mappings can be interpreted consistently with the radial model elaborated on the basis of AZIN2-LacZ material, and occasionally offer additional data of interest, particularly with respect to potential tangentially migrated cell populations. We’ll basically point out the elements of the model that express significantly these markers (Fig. 8). These preparations were both cut at an “amygdalar radial plane” oriented at 45 degrees relative to the entorhinal reference plane.

At perinatal stages (E18.5), *Lhx9* selectively labels strongly the rounded BMA nucleus, which represents the intermediate stratum component of our *anterior* radial unit (BMA; Fig. 8c–e); in the sections through the deepest part of BMA we observed its continuity with a less strongly labelled, small conic process, which adheres to the neighboring mass of the classic BLA nucleus (BMA; Fig. 8a–d). We interpreted this conic part as a remnant of the earlier periventricular stratum, now essentially disappeared. Otherwise, the periventricular amygdala appears uniformly negative for *Lhx9* at this stage. Superficially to BMA, and slightly separated from it by a cell-poor gap, appears a stratum of more dispersed *Lhx9*-positive cells, representing the conventional ACo nucleus (ACo; Fig. 8c–g). Remarkably, abundant *Lhx9*-labelled cells extend rostralward from ACo into AA, fully surrounding the *Lhx9*-negative NLOT nucleus (AA, NLOT; Fig. 8a–c). Cells of similar characteristics, which also are continuous with elements found in AA and ACo, extend medialwards into the *anteroventral medial amygdala nucleus,* as well as into the *posteroventral medial amygdala nucleus* (MeAV; MePV; Fig. 8a–f). Next to them there appears superficially, associated to the accessory olfactory tract, the strongly labelled BAOT nucleus (BAOT; Fig. 8d, e). Its different profile in this regard compared to the NLOT is remarkable. We think that *Lhx9*-positive neurons labelled within AA, MeAV, and MePV may represent elements developmentally related to (i.e., possibly originated in) the *anterior* amygdalar radial unit, which first migrated radially into the ACo, and from there advanced tangentially into the anterior and medial amygdala. BAOT may be the result of a different developmental process (its molecular profile is singular; Suppl. Table 1).

In addition, our *Lhx9* material uniformly showed laterally, separately from the *anterior* radial unit, an isolated positive periventricular patch deep to the amygdalopiriform area and adjacent to the BLP, which extends rostralwards towards the superficial area occupied by the PLCo; this formation clearly corresponds topographically to the retroendopiriform nucleus (REP), which we previously identified laterally to the BLP in AZIN2-LacZ material (REP; Fig. 8e, f). Its labelling with *Lhx9* is fully consistent with its AZIN2-LacZ image, displaying in particular its arch-like radial extension associated to the pallial amygdala (see Discussion). Importantly, it does not contact at any point the *anterior* unit derivatives. This selective *Lhx9*-labelling pattern, which supports its ascription as an additional pallial amygdalar radial unit, has no analogue at either the lateropallial dorsal endopiriform nucleus (EPD), or the ventropallial ventral endopiriform nucleus (EPV), both of which are found more rostrally under the Pir, each with a different genoarchitectonic pattern (Fig. 6).

Additional *Lhx9* reaction appears at the amygdalar *posterior* radial unit, at its periventricular (AHi) and intermediate/superficial (PMCo) strata. The expression within the periventricular AHi is strongest at its rostromedial subregion, found close to the dentate gyrus; this part shows a gradiental decrease of *Lhx9* signal lateralwards (AHiRM; Fig. 8e–g). The PMCoRM subregion is also strongly *Lhx9*-positive under the similarly labelled medial end of the AHiRM (PMCoRM; Fig. 8f, g); this expression disappears gradientally at the fuzzy border of PMCoRM with the rostrolateral and caudolateral subregions of the nucleus (Fig. 8g). *Lhx9* expression at both AHiRM and PMCoRM apparently coincides with the strong AZIN2-LacZ signal described above. In contrast, the corticoid cell layer of PMCoCL shows weak expression of *Lhx9* (PMCoCL; Fig. 8g).

The *Er81* expression pattern is more or less complementary to that of *Lhx9* in that it leaves essentially unlabelled the *anterior* radial unit. However, subtle differences are found, which recommend describing this marker separately. At the *lateral* radial unit, *Er81* expression is very weak at the LI nucleus, and the L nucleus remains strictly negative (LI; L; Fig. 8i–k). This is true as well for the corresponding CxAR superficial formation, in contrast with the positive CxAC subdivision belonging to the *basolateral* radial subunit (CxAR, CxAC; Fig. 8i–p). There is also intense *Er81* signal at the BLI and BLA (intermediate stratum) and a moderate expression at the BLP (BLI; BLA; BLP; Fig. 8i–p). As regards the *basomedio-medial* radial subunit, *Er81* strongly labels in a continuous fashion the periventricular BMPM and the intermediate BMIM nuclei; note the *Er81*-labelled BMPM again contacts the BLP via a slender rostrodorsal prolongation (passing above BMPL), but then rounds up medially to the weakly *Er81*-positive BMPL; the latter leads itself into the fully negative BMIL (BMIM; BMPM; BMPL; BMIL; Fig. 8l–p). The NLOT nucleus lying in the midst of the negative AA is massively *Er81*-positive, and relates likewise to the weakly *Er81*-positive cellular amygdalo-olfactory stream (AOS), which bridges the distance between the NLOT and the tip of the medial horn process of the BLA (NLOT; AOS; H; Fig. 8k–n). Finally, *Er81* labels rather strongly the rostromedial portion of the AHi, a member of the *posterior* radial unit, similarly as observed with AZIN2-LacZ and *Lhx9* (AHi; Fig. 8o, p).

### DiI experiments labelling radial glia in the pallial amygdala

**Fig. 9:**
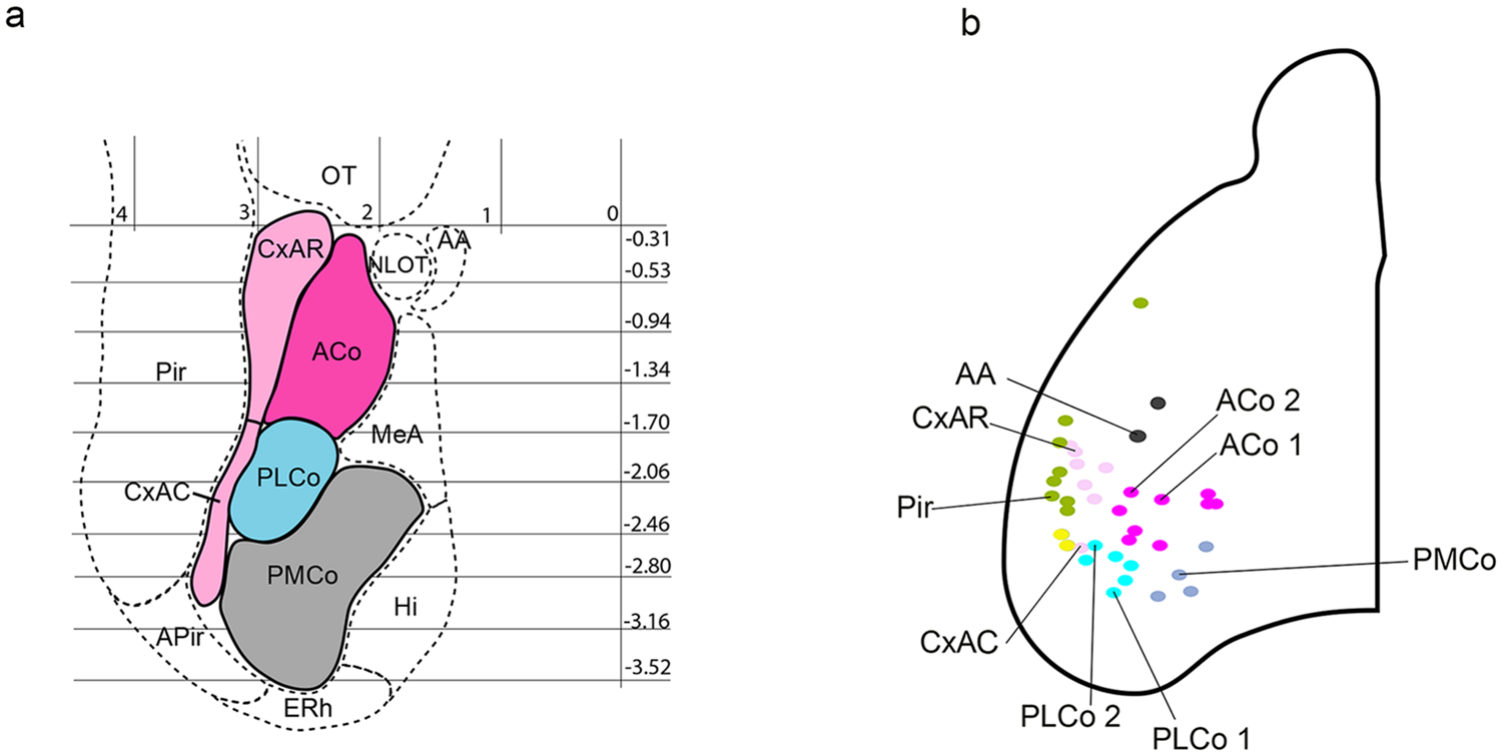
Graphic projection map of amygdalar cortical areas on the amygdalar surface and map of DiI injections. **a** Map of adult amygdalar cortical areas in horizontal plane projection according to Paxinos and Franklin (2001), as described in Material and Methods. **b** Map of DiI injection points at the amygdalar surface in E18.5/P0 mice, color coded according to areas in (**a**). The cases identified apart of the color-code by a specific tag are those shown in Fig. 10 (AA, Pir, CxAR and CxAC) and Fig. 11 (ACo1, ACo2, PLCo1, PLCo2 and PMCo)

To test the amygdalar radial relationships deduced from our gene mapping and structural analysis, we labelled amygdalar radial glia with DiI crystals inserted at the brain surface of fixed E18.5 and P0 mouse specimens. We used these stages, because glia immunoreaction often fails at more advanced perinatal stages (leading to a widespread unproven belief in the literature that radial glia cells cease to exist at these stages; however, this may be just a change in their immunoreactive epitopes). Moreover, architectonic development of the amygdala is sufficiently advanced perinatally to detect the major cell groups (particularly in combination with correlative gene patterns). Using the radial amygdalar section plane for analysis of the experiments, we checked in this way whether selected packets of radial glia cells could be followed from a mapped locus at the pial surface to a predicted ventricular end. To this aim, we first prepared a rough reference map of the adult mouse amygdalar brain surface by means of graphical projection upon the stereotaxic horizontal plane of the relevant superficial structures identified in coronal sections by Paxinos and Franklin (2001; Fig. 9a). Subsequent systematic anatomic classification of the sites where DiI crystals were found to be inserted after sectioning allowed us to progressively adapt the adult reference map to a perinatal map (Fig. 9b). The latter allowed us to choose the labelling sites more efficiently. It was found that the bulging olfactory tuberculum and the lateral olfactory tract (both visible in the fixed brains) also were useful orienting landmarks. The labelled positions were nevertheless always re-evaluated subsequently in the sectioned material of every case, after DAPI counterstain.

**Fig. 10:**
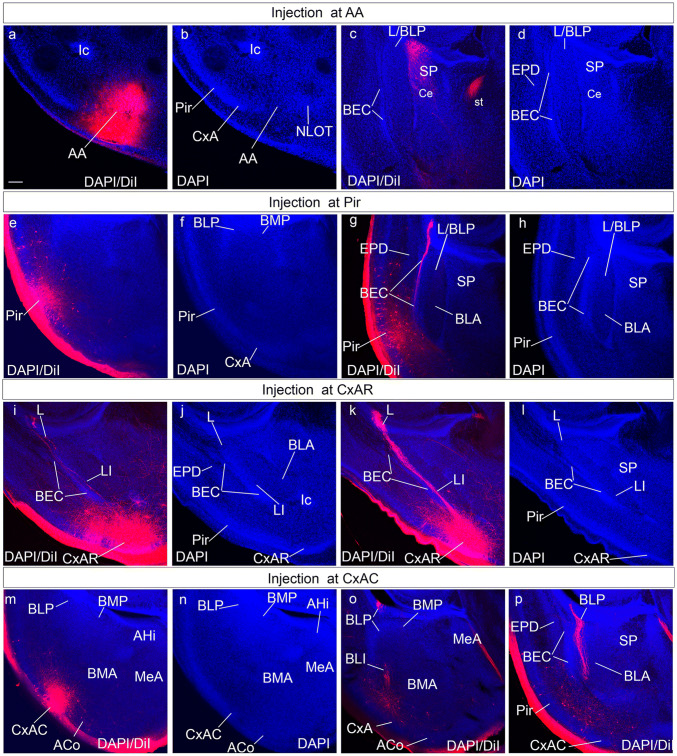
Radial glia DiI experiments in the amygdala region: AA, Pir and CxA. These panels illustrate several cases of pial DiI labelling experiments in mouse embryos at stages E18.5-P0; after fixation. the brains were sectioned in the amygdalar radial plane and counterstained with DAPI. **a**–**d** Confocal micrographs of a DiI injection at AA, at two caudorostral levels: (**a**, **b)**, show the same caudal DAPI-counterstained section with DiI fluorescence or without, for cytoarchitectonic reference; note the labelling site in **a**. **c**, **d** both show a more rostral section visualizing DiI fluorescence or not (only DAPI); note ventricular subpallium labelling in **c**. **e**–**h** Confocal micrographs of a DiI injection at Pir, at two caudorostral levels; **e**, **f** show the same caudal DAPI-counterstained section with DiI fluorescence (showing the Pir labelling site) or without, for cytoarchitectonic reference; **g**, **h** represent the same rostral level with DiI fluorescence visualization or not, illustrating radial glia labelling along the ventropallial BEC population into the extra-amygdalar ventral pallium neuroepithelium (**g**). **i**–**l** Confocal micrographs of a DiI injection at CxAR, in caudo-rostral order. **i**,** j** both represent the same caudal level with DiI fluorescence visualization or not (labelling site in **j**); **k**, **l** represent the same rostral level with DiI fluorescence visualization or not, illustrating in **k** radial glia labelling along the Li nucleus into the periventricular L nucleus. **m**–**p** Confocal micrographs of a DiI injection at CxAC, in caudo-rostral order: **m**, **n** both represent the same caudal section either with DiI fluorescence visualization or without (injection side in **m**). **o**,** p** show two more rostral sections illustrating labelled radial glia fibres penetrating past the BLA into the BLP. Scale bar represent 200 µm

**Fig. 11:**
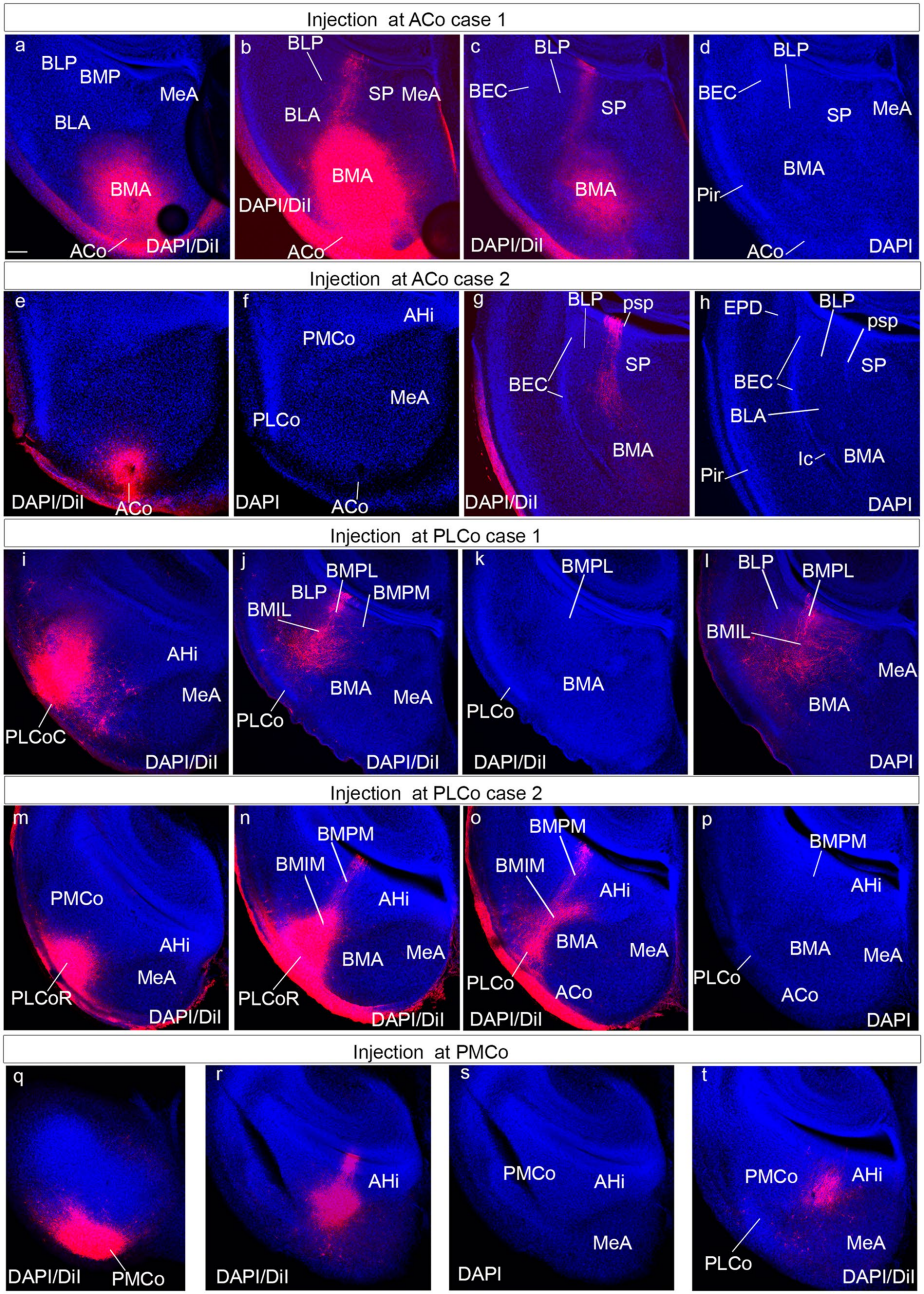
Radial glia DiI experiments in the amygdala region: ACo, PLCo and PMCo. These panels illustrate several cases of pial DiI labelling experiments in mouse embryos at stages E18.5-P0; after fixation. The brains were sectioned in the amygdalar radial plane and counterstained with DAPI. **a**–**d** Confocal micrographs of a DiI injection at ACo, case 1, at three caudorostral levels: **c**, **d**, show the same DAPI-counterstained section with DiI fluorescence or without, for cytoarchitectonic reference. Note the labelling site in **a** and the ventricular labelling in **b**, **c**. **e**–**h** Confocal micrographs of a DiI injection at ACo, case 2, at two caudorostral levels: **e**, **f** show the same DAPI-counterstained section with DiI fluorescence or without, for cytoarchitectonic reference; note the labelling site in **e**. **g**, **h** represent the same level with DiI fluorescence visualization or not; note the ventricular labelling in **g**. **i**–**l** Confocal micrographs of a DiI injection at PLCo, case 1, at three caudorostral levels: **j**, **k** show the same DAPI-counterstained section with DiI fluorescence or without, for cytoarchitectonic reference. Note the labelling site in PLCoC in **i**. Note the BMPL ventricular labelling in **j**,** l**. **m**–**p** Confocal micrographs of a DiI injection at PLCo, case 2, at three caudorostral levels: **o**, **p** show the same DAPI-counterstained section with DiI fluorescence or without, for cytoarchitectonic reference. Note the labelling site in PLCoR in **m**. Note the BMPM ventricular labelling in **n**,** o**. **q**–**t** Confocal micrographs of a DiI injection at PMCo, at three caudorostral levels: **r**, **s** show the same DAPI-counterstained section with DiI fluorescence or without, for cytoarchitectonic reference. Note the labelling site in PMCo in **q**. Note the BMPM ventricular labelling in AHi in **r**. Scale bar represent 200 µm

The insertion of a small DiI crystal was performed on recently fixed brains at E18.5 and P0 (*n* = 38 injections; summarized and color-coded in Fig. 9b). The labelled brains were kept in fixative solution in the dark for 2 weeks at 37 °C, before embedding them in 4% low melting point agarose, and radially oriented sectioning. After DAPI-staining, which provided some cytoarchitectonic references towards anatomic analysis, we studied the course of the single DiI-labelled glial bundle through the intermediate and periventricular amygdalar nuclei, as well as the relative topography of its ventricular end. The carbocyanine dye normally diffused efficiently from the glial endfeet located at the brain surface to the cell somata in the ventricular zone (Voight 1989; Miyata et al. 2001). We considered results valid only when a single isolated radial glia bundle was labelled, extending from the pial surface to the ventricular zone, irrespective of other labelling results occasionally present near the brain surface (the latter depend on the depth at which the DiI crystal was inserted, which did not affect local glial labelling). Collateral anterograde or retrograde labelling of neurons or axons was considered irrelevant for our purposes (e.g., sometimes the stria terminalis or the lateral olfactory tracts were labelled).

Control experiments placed at the AA, assumed to be subpallial in nature (AA and olfactory tubercle; *n* = 2), clearly labelled glial bundles ending at the ventricular zone of the striatal amygdala, after crossing the central amygdalar nucleus in their course (Ce; Fig. 10a–d). In other control experiments we inserted DiI crystals at the piriform and amygdalopiriform cortex, at different rostrocaudal levels (Pir *n* = 8; APir *n* = 2). In all these cases, the labelled radial glia bundles coursed radially from Pir or APir through the ventropallial migration stream found just lateral to the amygdalo-cortical boundary, reaching the cortical ventropallial ventricular zone (Figs. 1d, g, 10e–h). The ventropallial migratory stream reportedly involves at E18.5 persistent radial migration of neurons into the olfactory cortex, or the related deep ventral endopiriform nucleus (Puelles et al. 2016a, b); this stream coincides with a ventropallial packet of cortical radial glia processes (Fig. 1d, e). The correlative ventropallial cell stream (Fig. 1g, h) was previously identified by Medina et al. (2004), and related ulterior remnants of the ventropallial migration form the bed nucleus of the external capsule (BEC; Puelles 2014; Puelles et al. 2016a, b). We thus established the rostromedial (subpallial) and laterocaudal (cortical) edges of the pallial amygdala. These glia-labelling results imply that neither AA, Pir or APir cover superficially the amygdala, and thus represent neighbour cortical formations outside the pallial amygdalar radial domain (see “Discussion”).

We next aimed DiI crystals at the cortico-amygdaloid transition area (CxA; *n* = 7) testing different rostrocaudal levels, due to our previous data suggesting distinct rostral and caudal components (CxAR, CxAC). Radial glia bundles labelling rostral CxA passed through LI and bent caudalwards into the L (i.e., they had to be followed across more than one section); however, some of these markings reached L within a single section (Fig. 10i–l). DiI crystals placed at the caudal CxA instead marked a somewhat more medial glial bundle, which passed through the BLI and BLA and after a bend reached the ventricle across the BLP (Fig. 10m–p). These results are consistent with our amygdalar ascription of the CxA formation, with CxAR associated to the *lateral* radial unit and CxAC to the *basolateral* radial subunit.

The anterior cortical nucleus (ACo) is a rather extensive superficial pallial amygdalar area located medial to CxA, rostral to PLCo, and roughly caudal to the AA level occupied by the NLOT nucleus (Fig. 9a). We inserted various DiI crystals in this structure (ACo; *n* = 9) and uniformly followed the labelled radial glia bundles into the BMA and its small deep conic horn medially adjacent to BLA. From there the glial fibres slipped along the medial side of the BLA and L nuclei, reaching a narrow ventricular domain which bounds them from the central and medial amygdalar subpallium (globally, these diverse tracings are consistent with the approximately reconstructed locus marked in red in Fig. 5). At periventricular levels, this cryptic locus appears first medial to the L (rostrally) and then medial to the BLP and neighbouring dorsal part of BMPM (next to the palliosubpallial border at Ce and MeA levels; Figs. 5b, c, 11a–h). The ACo accordingly appears radially related to the *anterior* radial unit as defined above, but *not* to the *basal* radial unit, as was assumed in all previous accounts in the literature.

Next, we placed DiI crystals at the posterolateral cortical nucleus (PLCo, *n* = 6; Fig. 11i–p). Caudally placed PLCo crystals identified a radial glia packet passing through the BMIL into the BMPL (Fig. 11i–l). DiI crystals placed within rostral PLCo labelled instead radial glia extending through BMIM into BMPM (Fig. 11m–p). These data corroborate that the PLCo is superficially related to the two BMP (*basomedial*) radial subunits distinguished by differential AZIN2-LacZ expression. In contrast, when we labelled the superficial posteromedial cortical nucleus (PMCo; *n* = 4; Fig. 11q–t), the DiI-labelled fibres extended through the intermediate PMCo stratum into the AHi ventricular zone, caudomedial to the *basal* radial unit (Fig. 11q–t). This course clearly corresponds to the *posterior* radial unit. Due to its small size, we apparently did not hit the REPCo, so that we never saw a PLCo-related glial bundle targeting the periventricular REP laterally to the BLP.

### Allen atlas gene expression pattern analysis in the pallial amygdala

To expand the molecular characterization of our radial amygdalar units we studied 81 genes selected empirically for their differential adult expression in various architectonic elements of the pallial amygdala (apart of a few results from our own mouse ISH material, most data were taken from the Allen Developing [or Adult] Mouse Brain Atlas at P56). The consensus (E.G-C; L.P) expression of these markers across all amygdalar nuclei defined in our study was tabulated in an Excel sheet (Suppl. Table 1); in this Table the structures were grouped into postulated radial units and strata, for easier comparison between their components, and the genes were listed alphabetically. We included some neighboring non-amygdalar structures, for comparison. A rough analysis of this complex mapping already indicates the existence of many differential molecular patterns as well as some shared patterns both between the radial units and across their individual nuclear elements.

**Table 1:**
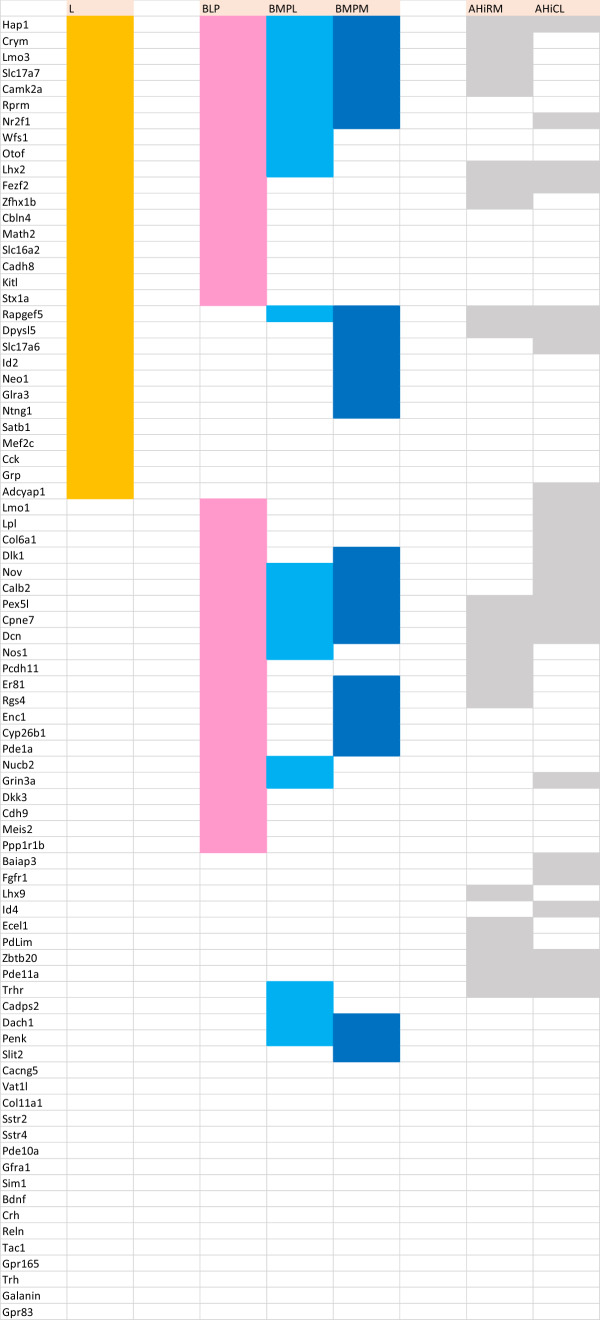
Expression similarity map of 81 gene markers across the periventricular stratum of the radial amygdala model

Nuclei studied: L, BLP, BMPL, BMPM, AHiRM and AHiCL. Color coded entries according to Fig. 2 for positive expression at the specified loci. Note the lines of Excel containing the data for each gene were regrouped empirically (differently than in Tables 2 and 3) to visualize optimally the similarity or lack of similarity between individual periventricular nuclei, compacting their expression codes over this gene list. Genes at the bottom are not expressed in the structures examined

**Table 2:**
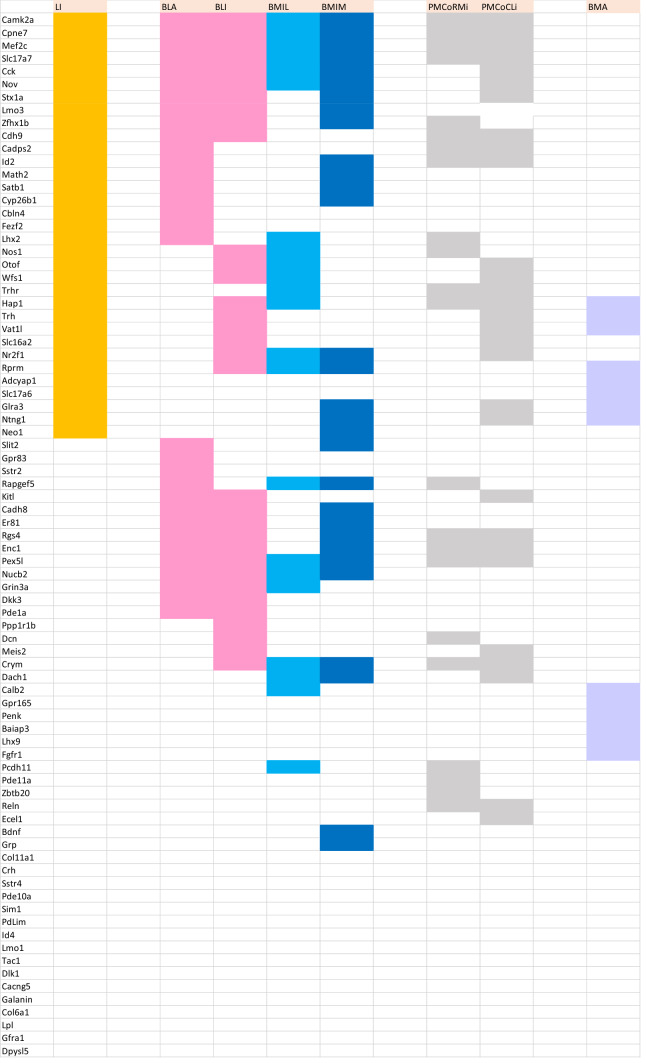
Expression similarity map of 81 gene markers across the intermediate stratum of the radial amygdala model

Nuclei studied: LI, BLA, BLI, BMIL, BMIM, PMCoRM, PMCoCL, BMA. Color coded entries according to Fig. 2 for positive expression at the specified loci. Note the lines of Excel containing the data for each gene were regrouped empirically (differently than in Tables 1 and 3) to visualize optimally the similarity or lack of similarity between individual intermediate nuclei, compacting their expression codes over this gene list. Genes at the bottom are not expressed in the structures examined

**Table 3:**
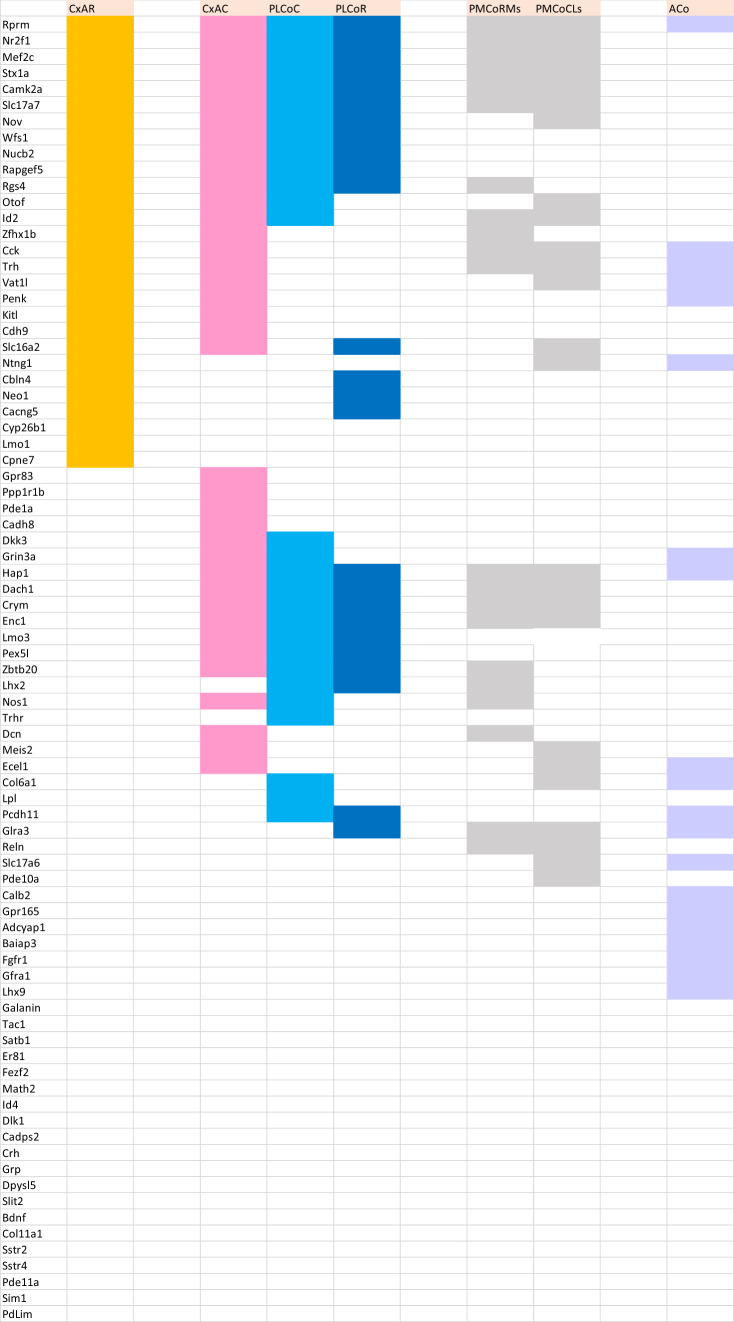
Expression similarity map of 81 gene markers across the superficial stratum of the radial amygdala model

Nuclei studied: CxAR, CxAC, PLCoR, PLCoC, PMCoRMs, PMCoCLs, ACo. Color coded entries according to Fig. 2 for positive expression at the specified loci Note the lines of Excel containing the data for each gene were regrouped empirically (differently than in Tables 1 and 2) to visualize optimally the similarity or lack of similarity between individual periventricular nuclei, compacting their expression codes over this gene list. Genes at the bottom are not expressed in the structures examined

**Table 4:**
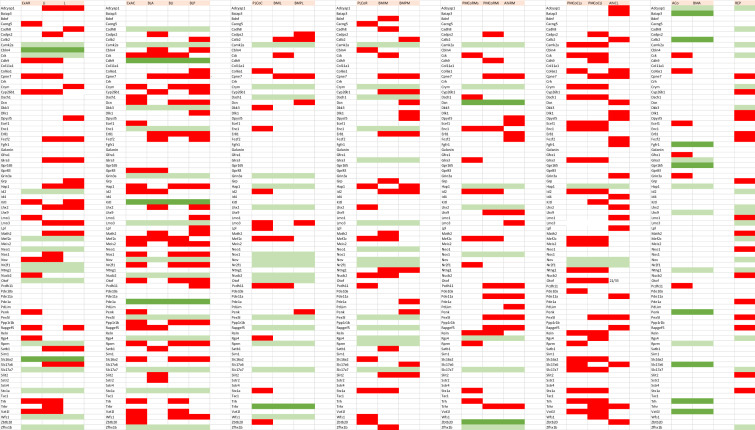
Unit-wide homogeneity map of 81 gene markers over the radial units postulated in the radial amygdala model

This Table is basically a copy of Suppl. Table 1 in which the red entries for markers which happen to label all three strata of any amygdalar unit were changed into either light or dark green (we called these patterns *unit-wide*). The color code for genes labeling only one or two strata of the units remained red. The unit-wide genes entered in light green were not completely selective, since they in repeated their expression in two or more radial units. On the other hand, a subgroup of unit-wide genes were fully selective in being present only in one of the radial units; there cases were entered in dark green. Numeric results about these patterns are presented in Table 5

**Table 5:** Quantification of unit-wide homogeneity analysis

	A	B	C	D	E	F
	No. of genes +	All green	% B/A	Dark green	% C/A	% C/B
L	44	15	34	2	4.54	13.33
BL	55	16	29	3	7.2	25
BML	34	17	50	1	2.94	5.82
BMM	45	14	31	0	0	0
PRM	36	10	28	2	5.55	20
PCL	49	2	6	0	0	0
A	20	14	70	8	40	57.4
REP	32	16	50	0	0	0
Total	*T* = 81					

Though we cannot know with any certainty whether the 81 studied gene patterns are representative of the complete diversity of expression patterns found in the mouse pallial amygdala, we were ourselves impressed by some quantitative differences observed among our data reported in Table 4, which were evaluated in Table format as follows. We counted first in column **A** how many genes of the total (81) were expressed in at least one component of the diverse radial units (note the list of radial units at left lacks the PRL subunit; this was due to lack of detailed data about it in our Suppl. Table1, because we were not considering the existence of this subunit when that Table was elaborated). This number of expressed genes ranges between 20 and 55, but refers to qualitatively different genes in all radial units, as shown in Tables 1, 2, 3 (similarity analysis). If we should assume arbitrarily that the molecular profile of any radial amygdalar unit involves a combination of 100 genes expressed either partially or unit-wide, then column **A** would measure how far we would be from knowing such profiles. However, it can be that such codes require different numbers of genes for different units, in which case such assessment is less informative. Column **B** indicates how many unit-wide gene patterns (light and dark green entries together) were found in each unit. Interestingly, all units had such patterns, ranging from 2 in PCL) to 17 in BML). Column **C** gives the percentages of items in B relative to the numbers of expressed genes given in A. The C data range widely between 6% (PCL) and 70 (A). These data seem remarkably high on the whole, suggesting that perhaps our expert selection of 81 genes out of 4000 in the Allen Developing Mouse Brain database (and some 20.000 in the genome) succeeded in identifying many of the genes that constitute relevant molecular profiles in the region of interest. Column **D** gives the numbers of selective unit-wide gene patterns) per radial unit (dark green in Table 4. Here the numbers drop significantly, to the point that some radial units are left without such patterns, and others have only 1 or 2. Nevertheless we still see 8 selective unit-wide genes at the *anterior* unit, and 3 at the *basolateral* unit. The percentage relative to expressed genes is given in column **E** (where we see that 40% of genes expressed at the *anterior* unit are selectively unit-wide). Finally, column **F** gives the percentage of selective unit-wide genes (column **D** data) relative to all unit-wide genes (column **B**). Here we see again surprisingly high percentages at the *basolateral*, *basomedio-lateral* and *anterior* units (57% in the last case)

We then extracted from the primary Suppl. Table 1 separate *molecular profile similarity maps* for the sets of periventricular, intermediate and superficial amygdalar elements (Tables 1, 2, 3, respectively). To this end, the gene patterns obtained for each stratum (note that not all 81 genes were expressed in all the strata) were copied from Suppl. Table 1 into a separate Excel sheet. The individual lines carrying information from each marker were reordered empirically over iterated intermediate stages, progressively joining the lines according to their closest pattern of similarity. This procedure aimed to visualize optimally by their distinct spatial grouping the shared or non-shared expression properties of the diverse genes. These maps empirically highlight in a transparent graphic format gene data suggesting similar versus dissimilar relative distributions of several dozens of genes within each stratum. The predicted and observed result was that the stratum-specific nuclei corresponding to *different* postulated radial units have clear-cut *differential combinatorial molecular profiles*, irrespective of a number of shared expression properties. This result was verified for the three strata (Tables 1, 2, 3). Of course, other slightly variant *similarity mappings* are perfectly possible, by alternative rearrangement of the individual gene lines in the table, without altering the final conclusion.

To examine these gene patterns from a different viewpoint, we separately visualized (again alphabetically) in Table 4 what we define as *unit-wide homogeneity maps*. This concept refers to our mining of those rarer gene patterns which are shared through all three strata of the postulated radial units (thus apparently providing a sort of shared or common molecular identification of all or most derivatives of individual radial units). To this aim we marked the unit-wide positive cases in light *versus* dark shades of *green* in Table 4, compared to alternative genes marked in *red*, indicating more selective genes, which label the radial units only partially. Light green markings are more abundant, and often characterize jointly 2 or more radial units (i.e., the same gene is shared by *all strata* of *several* radial units); dark green markings identify those genes whose labeling of *all strata* is restricted to *a single* radial unit (Table 4). In Table 5 we analyze numerically and percentually for each unit the proportion of unit-wide positive genes compared to either the full list of 81 genes studied (green entries-column B-relative to the sum of green, red and negative entries), or, more relevantly, relative to the total number of genes expressed in each radial unit (column A-that is, proportion of green entries relative to sum of red and green entries-column C). The numbers of unit-wide homogeneous markers oscillated between 2 and 16 across the radial units (6–70% in terms of expressed genes; Table 5, columns B, C). The strongest or most distinct profile corresponds to the *anterior* unit.

While *unit-wide homogeneity maps* in general highlight combinatorial patterns suggesting a sort of common molecular identity code for the whole units, many individual unit-wide genes were found to have this role in two or more radial units, being thus less-specific homogeneous characters. In Table 4 these cases were identified in *light green* color. In contrast, *dark green* entries were used for specific unit-wide genes which were *uniquely* present in a particular unit. The latter more restrictive selection left us with the *basolateral and anterior* units still retaining significant numbers of selective unit-wide markers (3 and 8, respectively; Table 5, column D; see percentages in columns E and F), whereas other units showed a much lower number of such markers (1, 2), or none. Since the total pool of operational genes available in the developing mouse amygdala surely is much larger than our *ad hoc* list of 81 markers, the quantitative details may change significantly in more inclusive approaches. We nevertheless submit that our partial gene expression analysis has some significance in support of our model. Our approach could have identified a majority of gene patterns uniformly distributed across given strata of the pallial amygdala units, or an absolute lack of selective molecular sharedness within the stratified radial complexes postulated, but this did not occur.

We also collected from the Allen Adult or Developmental Mouse Brain Atlases a gallery of representative images selected from the gene expression patterns interpreted in Suppl. Table 1. It should be kept in mind that Allen atlas coronal sections are oblique by some 45 degrees to our radial amygdalar sections. A selection of these images is shown in Suppl. Fig. 1 to witness our interpretive criteria (‘what is exactly what we call this or that’), as well as the existence of real gene patterns which are imitated by our morphological interpretations in the model. The considerable agreement observed is not due to casual success, but resulted from our repeated efforts to adapt our thinking over time to the observed data (we conceived successive alternative interpretations of the radial model, and gradually perfected them until good results were obtained all around).

## Discussion

We grouped mouse pallial amygdalar structures into radial histogenetic domains, each possessing deep, intermediate and superficial nuclei and separate skeletons of radial glia. We expected some new conclusions to emerge, particularly about the complexity of the intermediate stratum, since conventional amygdalar schemata do not resolve this task (i.e. they offer different numbers of nuclei in each stratum). Our correlative analysis of gene markers showed that the postulated radial units correlate with distinct molecular profiles (codes), which theoretically must result from differential patterning. Since the pallial amygdala is radially independent of the cortical pallial field (Puelles et al. 2019; present data), we left aside the issue of pallial sectors which may participate in the amygdala (Medina et al. 2004, 2017; Tole et al. 2005; Remedios et al. 2004, 2007; Puelles et al. 2016a; Ruiz-Reig et al. 2018). We will touch this issue in a companion analysis in preparation dealing with planar amygdalar relationships.

Literature on amygdalar neuroanatomy in rodents is based on conventional coronal and horizontal sections. The former intersect the amygdalar region roughly 45 degrees oblique to its radial structure as assessed by glial immunostaining (e.g., Remedios et al. 2007; present results). Typically, standard pallial amygdalar nuclear names (e.g., De Olmos et al. 2004; Paxinos and Franklin 2013; Paxinos and Watson 2014; Olucha-Bordonau et al. 2015) describe as ‘anterior’ various *superficial* or *intermediate* elements (e.g., **A**A, **A**Co, BM**A**, or BL**A**), and call ‘posterior’ some periventricular elements (e.g., BL**P**, BM**P**), but not others (L, or AHi). This standard or historical approach is not clarifying from a developmental or comparative viewpoint. We departed from tradition using sections cut in a novel *amygdalar radial* section plane ranging between 45° and 30° relative to standard coronal sections (“Materials and methods”). We *explicitly* contemplated both ventricular and pial surfaces as they relate to amygdalar radial structure (Figs. 2, 3).

It might be asked why a model of the radial structure of the mammalian pallial amygdala is of interest. It probably does not contribute much to hodological or functional lines of research, though it arguably offers a more complete and thus *better* anatomic map of the subdivisions of this system, which should be preferred for all purposes (e.g., our division of classic BMP into distinct BMPL and BMPM parts and corresponding distinct novel radial complexes maybe merits some hodological and physiological attention). Our model thus implicitly poses the need to reexamine all previous hodological and functional conclusions. We also think that a better, more gene-based model of the pallial amygdala has unforeseen consequences in other directions. For instance, we hold that *causal developmental* and *evolutionary analysis* of the amygdala *requires* specific attention to our radial complexes (or to eventual perfected *radial models*), since these represent ontogenetic and evolutionary units of variation, as we argue next.

Given that the variety of radial complexes must result from earlier differential molecular patterning of the pallial amygdalar field of progenitors, each radial unit derives from a differentially specified progenitor subfield, and becomes across evolution an independent unit of developmental variation, in so far as differential field histogenesis and morphogenesis represent the relevant developmental constraints imposed upon genomic readout at the different areal positions of the amygdalar field. Radial complexes are developmental *units,* because they arise out of different unitary progenitor populations which are *homogeneous in their own molecular profiles and differential relative to their neighbours*. They accordingly also represent units of pattern. The pallial amygdala cannot be explained *causally* without considering these radial units and their respective differential histogenetic mechanisms as independent variables.

Since evolution occurs via changes in morphogenesis, amygdalar radial complexes taken as developmental units may have diverged over millions of years. The complexity of radial unit derivatives in terms of differential growth, neuronal typology, stratification, connectivity and synaptogenesis surely has changed subtly or markedly in different vertebrate lineages (we assume an early evolutionary origin of the earliest amygdalar primordium at least at the level of gnathostomes; a pallial amygdala has not been identified yet in agnathans; Nieuwenhuys and Nicholson 1998; Wicht and Nieuwenhuys 1998; Striedter and Northcutt 2020). As occurs in other brain regions, ancestral forms of the pallial amygdala may show less strata, less migration, less or different connections, less distinct neuronal types, etc., but possibly the *number* of radial units is relatively more resilient to evolutionary change, due to its root in patterning mechanisms and the possible necessity of a complete radial organisation to satisfy functional (i.e., adaptive fitness) requirements (Nieuwenhuys and Puelles 2016). Comparative thinking will be helped by searching in amygdalar studies topologically comparable and molecularly distinct histogenetic fields, taken as field-homologous units (Puelles and Medina 2002), and then try to explain the different results obtained in the anatomic analysis of the adult mantle layer across species.

Planar patterning mechanisms, such as morphogen gradients, are likely to act *orthogonally* to the radial complexes in the plane of the amygdalar neuroepithelium. The fact that such patterns become absolutely cryptic in arbitrary section planes oblique to the primary radial glial structure possibly explains why there is a remarkable dearth of patterning hypotheses on the pallial amygdala, while considerable progress characterizes our present understanding of the possibly more complex patterning of the cortical pallium. Our present radial model should thus help in visualizing morphogen sources and both topologic directions and extent of gradiental morphogen signals, not only in the mouse, but also comparatively in diverse vertebrate lineages. The implicit structural postulates of the model should improve the design and analysis of experimental studies, aiding, for instance, the interpretation of transgenically-engineered changes of phenotype (gain- or loss-of-function experiments).

### Our amygdalar model

The pallial amygdalar structural schema reflected in our radial model first resulted in an early approximation from our examination of adult and perinatal cell populations in AZIN2-LacZ, *Lhx9* and *Er81* genoarchitectonic material sectioned in the amygdalar radial section plane. The radial units postulated in this model were shown in most cases (no data for *rep*) to be consistent with parallel experimental labelling results on radial glia. Correlation with the expression patterns of some 80 gene markers further corroborated and perfected the general details of the proposed radial structure.

The observed and variously tested radial structure of the mouse pallial amygdala consists in 5 fundamental radial units (*lateral, basal, anterior, posterior* major domains plus the extra *rep* formation; Fig. 2). The *basal* and *posterior* radial units are each subdivided molecularly into three subunits which extend across all three strata. There results a two-level classification (units and subunits) which is convenient, because the four major units correlate with the four well-known superficial corticoid formations (CxA, PLCo, ACo, PMCo, respectively), as well as with classical periventricular or intermediate nuclei (L, BLP + BMP, BMA, and AHi, respectively).

Such results, and particularly the distinction of novel intermediate components, have some impact upon the standard schema and names of pallial amygdalar nuclei, implying a subtly modified classification. There are novelties in the definition and naming of individual nuclei, particularly of the intermediate stratum. The latter was not well understood by classic authors, who used less discriminative methods. We showed that novel notions such as LI, CxAC, BLI, BMIM, BMIL, and REPI are both realistic and necessary (Suppl. Fig. 1; Tables 1, 2, 3, 4; Suppl. Table 1).

The newly postulated *rep* radial complex (encompassing the radially expanded REP, which was conventionally seen only as a smaller population ascribed to a deep stratum of the periamygdalar piriform cortex, plus the old intermediate BLV cell group, now renamed REPI, and the superficial REPCo) is clearly distinct from the other radial units in topography, as well as in molecular terms (Tables 1, 4, 5). We think that the REP’s AZIN2-LacZ-revealed new *radial* image cannot be interpreted as an allocortical (Pir) *layer*. It lies at the border of the amygdala with the APir part of the olfactory allocortex, adjacent to BLP. Alternatively, the REP might be ascribed to the APir, but we think that its radial characteristics (Fig. 2d) are difficult to explain within the allocortex schema.

While resolving the ascription of intermediate nuclei and superficial structures to the periventricular elements some unexpected aspects emerged. For instance, glial and molecular evidence accrued indicating that the conventional BMA nucleus does not connect radially with the periventricular BMP nucleus, as is widely assumed in previous literature (and we initially assumed as well). Our partial developmental analysis of *Lhx9* expression, which selectively underlines this complex, jointly with contrasting early E12.5 expression data recorded in the literature (Remedios et al. 2004; Medina et al. 2017; Garcia-López et al. 2008), suggested that cells in this radial unit arise separately from the BMP and translocate massively radially into the *anterior* intermediate and superficial strata (BMA, ACo), leaving only glial processes at their ventricular origin; this last point was verified experimentally (Figs. 2c–e, 11a–h).

The existence of superficial cells contiguous to the BMA/ACo population which apparently may have translocated tangentially into the subpallial anterior and medial amygdala was another surprise (Fig. 8a–g). We have some evidence for the start of such translocations already at E13.5, consistently with the early birthdates of all these superficial cells (unpublished data to appear in a companion paper in preparation). This interpretation retains the conventional concept of AA as an amygdalar part of subpallium.

Secondly, our study of the large intermediate BLA nucleus (which had never been associated to any amygdalar corticoid formation, but was correctly associated to the BLP) indicated that its radial connection with a novel superficial entity (CxAC) was mediated by a previously undescribed intermediate BLI cell population. The latter’s bent radial continuity into the ventricle interests essentially the *lateral halves* of BLA and BLP (Figs. 2g, 8h). There is in addition a medial part of BLA which includes cells of similar typology and molecular profile, expressing differentially AChE. These diverge in medial direction from their natural radial unit, forming first a cap over the rounded BMA nucleus and then a medially protruding ‘horn-like’ process that protrudes into the MeA (H; Fig. 8h). The histochemical AChE data on BLA found in several rodent atlases suggest that the lateral and medial parts of BLA form an unified whole, but we also noted that TH immunoreaction appears preferentially in the lateral part of BLA (Fig. 5d).

We discovered that the well-known lateral nucleus (L), which lies periventricularly, but is not conventionally associated to any intermediate or superficial amygdalar components, can indeed be radially related to specific intermediate and superficial structural elements. We identified the corresponding intermediate population as the novel LI nucleus (previously ascribed to the external part of BLA), and pinpointed the superficial element as our CxAR (Fig. 2f).

We found that the conventional intermediate BLV nucleus was radially unrelated to BLA, and it does not show either AChE or TH expression (characteristic BLA markers). It belongs instead as an intermediate stratum component (REPI) to the new radial unit underlined by strong AZIN2-LacZ and *Lhx9* signals (among others), which also includes the periventricular *retro-endopiriform* nucleus (REP) and a novel superficial mass (REPCo) found rostral to the classic PLCo (Fig. 2d). Since there is no evidence other than topographic vicinity that relates the classic BLV to the BLA, or any other part of the *basolateral* radial subunit, we considered that the usual ‘BLV’ name is incorrect and confusing, and should be changed for clarity to ‘intermediate REP’ (REPI).

Molecular and experimental glial results revealed that the radial complex ending superficially at the PLCo originates from the periventricular BMP. This radial stream appears divided into two molecularly distinct moieties or subunits (BMPL, BMPM), and imply corresponding two novel intermediate cell masses (BMIL, BMIM; Fig. 2a–c, e).

The *posterior* radial unit includes the AHi periventricular formation and the classic PMCo nucleus. The latter can be differentiated into an intermediate stratum and a superficial or corticoid stratum. Former attempts in rodent atlases to subdivide this complex into distinct topographic parts were corroborated in our material, but the suggested descriptors resulted somewhat contradictory semantically. We accordingly distinguished clear-cut *caudolateral*, *rostromedial* and *rostrolateral* molecularly distinct AHi/PMCo subdivisions. The *rostrolateral* component separates somewhat from the rest of the complex and ends instead of at the PMCo at its own thin RLCo superficial corticoid plate, found medially adjacent or attached to the PLCo. Many genes corroborate this pattern (an apparent bridge between AHi and PLCo).

### Radial and tangential migrations

Radial glial structure represents a topologic mapping of points of the ventricular surface upon corresponding points of the pial surface (Nieuwenhuys and Puelles 2016). Radial glia cells form the skeletal array of guides for standard neuronal radial migration; potentially this array is also used for neuronal or axonal stratification (insofar as the radial glial processes may be differentially decorated by adhesive epitopes at different points of their course, and used as references by neurons and axons). This glial radial array results deformed over time due to differential morphogenesis of the mantle layer, so that straight glial courses may become passively bent, compressed together, or fanned out at specific places. However, there is so far no evidence that growing radial glial processes ever violate the boundaries of the radial unit to which they belong (e.g., glial data on ventral and lateral pallium in Puelles 2014).

Available autoradiographic neuronal birthdate analyses on the mammalian pallial amygdala (notably Lammers 1976 in the Syrian hamster, and Bayer 1980 in the rat) support that superficial amygdalar populations are generally born earlier than intermediate and deep populations, a pattern consistent with an overall outside-in stratification. However, according to the present analysis, the *anterior* radial unit apparently is characterized by a singular more intense radial migration process, which leads to complete depopulation of its periventricular stratum, jointly with translocation of these deep cells to superficial positions (BMA and ACo; Fig. 8). Some of these superficial *Lhx9*-positive cells further may translocate subpially beyond the borders of the radial unit proper, and even of the pallial amygdala, entering the neighboring subpallium (AA and MeA).

Interestingly, the birthday data of Bayer (1980) on the ACo and BMA nuclei reveal oddly protracted periods of neurogenesis extending from E12 to E17 (ACo) and from E13 to E20 (BMA). These data on *late-born* superficial *anterior* unit cells (otherwise contradictory with the general outside-in pattern mentioned above) can be explained by our hypothesis of a massive radial migration at this radial unit; this hypothesis predicts that late-born periventricular cells should be found within the BMA/ACo and other (tangentially migrated) superficial derivatives of this radial unit.

Analysis of radially arranged mantle complexes can be complicated by superposed tangential migrations. Nevertheless, pervasive tangential migrations of subpallial interneuron contingents to the pallial amygdala (e.g., somatostatin-positive GABAergic cells; Puelles et al. 2016b; see also Hirata et al. 2009) apparently do not disrupt the basic radial pattern of the amygdalar nuclei, though they do not invade equally all the nuclei (*loc.cit*.). There were previously no suggestions of *pallio-pallial* amygdalar tangential displacements, if we leave apart the NLOT migration, which represents sequential tangential and radial displacements of the prospective layer 2 population of this nucleus along a particular path apparently enclosed finally within the subpallial amygdala, though with a still controversial pallial origin (Remedios et al. 2007; Puelles et al. 2019; present results; note in our Fig. 6 the relative distant positions of neocortex (dorsal pallium) and the amygdalar complex that forms the NLOT).

However, we presently conjecture that the whole medial part of the BLA nucleus including its horn translocates lateromedially atop the BMA nucleus (Fig. 8h). The BLA horn is selectively labelled in AChE-reacted atlas preparations (e.g., see Paxinos and Franklin 2013; their plates 42, 121, 138), thus confirming its typologic affinity with the BLA, also underlined by several of our gene markers. Near the BLA horn, but inside the amygdalar subpallium, there appears the novel *amygdalo-olfactory stream* (AOS), which is labelled differentially by the AZIN2-LacZ, *Er81* and *Cyp26b1* markers, among others not found in BLA (see Suppl. Table 1). The rather tenuous string of AOS cells apparently decorates the radial migration path of the NLOT layer 2 cells (Remedios et al. 2007), and ends at the NLOT layer 3, also forming a tenuous shell around the whole NLOT complex. It may be speculated that formation of the BLA horn (or of the whole medial BLA ‘cap’) and the AOS somehow responds to, or correlates with, the NLOT migration phenomenon.

The BLA nucleus is the most deformed radial part of the whole pallial amygdala, as is reflected also in its rostral glial knee-bend (Figs. 1b,c, 2g), as well as in the pattern of surrounding striatum-derived amygdalar intercalated cell aggregates (Ics). Selective *Meis2* labelling shows that Ics elements form a subpallium-derived net surrounding the intermediate parts of the *lateral* and *basolateral* radial units, separating them from the striato-amygdalar and central amygdala areas (subpallium; Suppl. Figs. 1k–m). We thus view Ics as the result of a discontinuous migration along the tridimensionally deformed striato-amygdalar pallio-subpallial boundary. This offers the first embryologic interpretation of the distribution of multiple intercalated nuclei within the mammalian amygdala (actually disposed along the topologic palio-subpallial surface).

### Differences in the molecular profile of defined amygdalar radial units

Tables 1, 2, 3, 4, and 5, as well as Suppl. Table 1 and Suppl. Fig. 1 summarize our analysis of 81 selected gene patterns expressed differentially in the amygdala (as found in the Allen Adult/Developmental Mouse Brain Atlases). The overall picture (Suppl. Table 1) shows great variability, including numerous shared or differential expression patterns. However, our molecular similarity analysis of the codes obtaining at individual amygdalar *strata* (Tables 1, 2, 3) showed that each of the radial units we postulate in our model has a characteristic *differential profile* of expressed markers, irrespective of the presence of shared genes. Moreover, analogous empiric analysis of *unit-wide molecular homogeneity* (i.e., genes shared across all strata of given radial units) showed a remarkable proportion of such patterns. This feature was most marked in the *anterior* radial unit, where up to 70% of the genes expressed (20 from our list of 81 markers) were distributed unit-wide (Tables 4, 5); curiously, this is also the unit which expresses *less genes* among our list of 81 markers, and the only one where deep stratum cells commingle in the adult with those of the intermediate and superficial strata (Table 5). Some unit-wide markers were shared in two or more distinct radial units (*n* = 31); of these, the majority (17) characterized two, often non-adjacent, radial units, whereas 5 appeared in 3 units, 6 in four units, 1 in 5 units and 2 in 6 units; note here we count the *basal* and *posterior* subunits separately). Leaving these multiple unit-wide cases aside left us still with a number of *unit-selective radially homogeneous genes* present only in a *single* unit (namely 8 of them *only* in the *anterior* unit*,* and 3 *only* in the *basolateral* unit*;* dark green markings, Tables 4, 5). We do not attach much significance to the apparent quantitative differences observed here between diverse radial units, due to the limited list of 81 genes selected for analysis. A more inclusive list of several hundred genes might have provided quite different relative numbers than our limited sample. See legend to Table 5. However, the qualitative conclusion remains that some genes do differentially delineate jointly all elements of some of the postulated radial units or subunits.

## Conclusions

Morphologic and histogenetic understanding of the pallial amygdalar complex seems to be improved by our present *radial amygdalar model*, wherein most classic subdivisions acquire enhanced meaning by means of subtle changes of concept and addition of needed details visualized by means of genoarchitectonic analysis and experimental labelling of radial glial organization. The model should be particularly useful for the analysis of amygdalar patterning, as well as for studies on amygdalar evolution, but also represents a motivation for more discriminative studies of connectivity and function. Due to the complex network of amygdalo-amygdalar interconnections, we do not expect specific functions associated to the radial units.

## Electronic supplementary material

Below is the link to the electronic supplementary material.

Supplementary file1 (PDF 1072 kb)

## Abbreviations

2: Layer 2
3: Layer 3
4: Layer 4
A: Amygdala
AA: Anterior amygdala
ac: Anterior commissure
ACo: Anterior cortical nucleus
AHi: Amygdalo-hippocampal area
AHiCL: Amygdalo-hippocampal area, caudolateral part
AHiRL: Amygdalo-hippocampal area, rostrolateral part
AHiRM: Amygdalo-hippocampal area, rostromedial part
AI: Insular cortex, agranular part
ant: Anterior radial unit
AOS: Amygdalo-olfactory stream
APir: Amygdalo-piriform area
ASt: Amygdalo-striatal transition area
Au1: Auditory cortex
BAOT: Bed nucleus of the accessory olfactory tract
bas: Basal radial unit
BEC: Bed nucleus of the external capsule
bl: Basolateral radial subunit
BLA: Anterior basolateral nucleus
BLI: Intermediate basolateral nucleus
BLP: Posterior basolateral nucleus
BMA: Anterior basomedial nucleus
bml: Basomedio-lateral subunit
bmm: Basomedio-medial subunit
BMP: Posterior basomedial nucleus
BMIL: Lateral intermediate basomedial nucleus
BMIM: Medial intermediate basomedial nucleus
BMPL: Lateral posterior basomedial nucleus
BMPM: Medial posterior basomedial nucleus
CA1: Field CA1 of the hippocampus
CA3: Field CA3 of the hippocampus
CeA: Central amygdalar nucleus
CeL: Central amygdalar nucleus, lateral part
Cl: Claustrum
cl: Caudolateral posterior radial subunit
CxA: Cortex-amygdala transitional area
CxAC: Cortex-amygdala transitional area, caudal part
CxAR: Cortex-amygdala transitional area, rostral part
d: Dorsal
DG: Dentate gyrus
EPD: Dorsal endopiriform nucleus
EPV: Ventral endopiriform nucleus
ER: Entorhinal cortex
ERhL: Lateral entorhinal cortex
ERhM: Medial entorhinal cortex
ERhV: Ventral entorhinal cortex
fi: Fimbria of hippocampus
GI: Insular cortex, granular part
GPe: Globus pallidus, external part
GPi: Globus pallidus, internal part
H: Horn of BLA
Hi: Hippocampus
Ic: Intercalated nucleus of the amygdala
ic: Internal capsule
l: Lateral
L: Lateral nucleus
lat: Lateral radial unit
LI: Intermediate lateral nucleus
m: Medial
MeA: Medial amygdala
MeAD: Medial amygdala, dorsal part
MeAV: Medial amygdala, ventral part
NCx: Neocortex
NLOT: Nucleus of the lateral olfactory tract
OT: Olfactory tuberculum
ot: Optic tract
ped: Peduncle
PRh: Perirhinal cortex
Pir: Piriform cortex
PLCo: Posterolateral cortical nucleus
PLCoC: Posterolateral cortical nucleus, caudal part
PLCoR: Posterolateral cortical nucleus, rostral part
PMCo: Posteromedial cortical nucleus
PMCoCL: Posteromedial cortical nucleus, caudolateral part
PMCoRL: Posteromedial cortical nucleus, rostrolateral part
PMCoRM: Posteromedial cortical nucleus, rostromedial part
PrS: Presubiculum
PoRh: Postrhinal cortex
post: Posterior radial unit
REP: Retroendopiriform nucleus
REPI: Retroendopiriform intermediate area
REPCo: Retroendopiriform cortical area
rep: Retroendopiriform radial unit
RL: Rostrolateral subdivision of the posterior radial unit
rl: Rostrolateral posterior radial subunit
rm: Rostromedial posterior radial subunit
Rt: Reticular nucleus, prethalamus
SP: Subpallium
St: Striatum
st: Stria terminalis
STh: Subthalamic nucleus
Th: Thalamus
v: Ventral
