# Supplementary material for: A radial histogenetic model of the mouse pallial amygdala
Source: Brain Struct Funct. 2020 Jun 24;225(7):1921–56. doi: 10.1007/s00429-020-02097-4 (PMC7473974; doi:10.1007/s00429-020-02097-4)
Supplement: Supplementary file 1 — Supplementary file1 (PDF 1072 kb) [file 429_2020_2097_MOESM1_ESM.pdf]

Supplementary materials

A radial histogenetic model of the mouse pallial amygdala

Elena Garcia-Calero[a], Margaret Martínez-de-la-Torre[a], Luis Puelles[a]

[a]Department of Human Anatomy, School of Medicine and IMIB-Arrixaca Institute, University of Murcia, 30120, Murcia, Spain

Corresponding author: Elena Garcia-Calero; e-mail: ecalero@um.es

Suppl. Fig.1

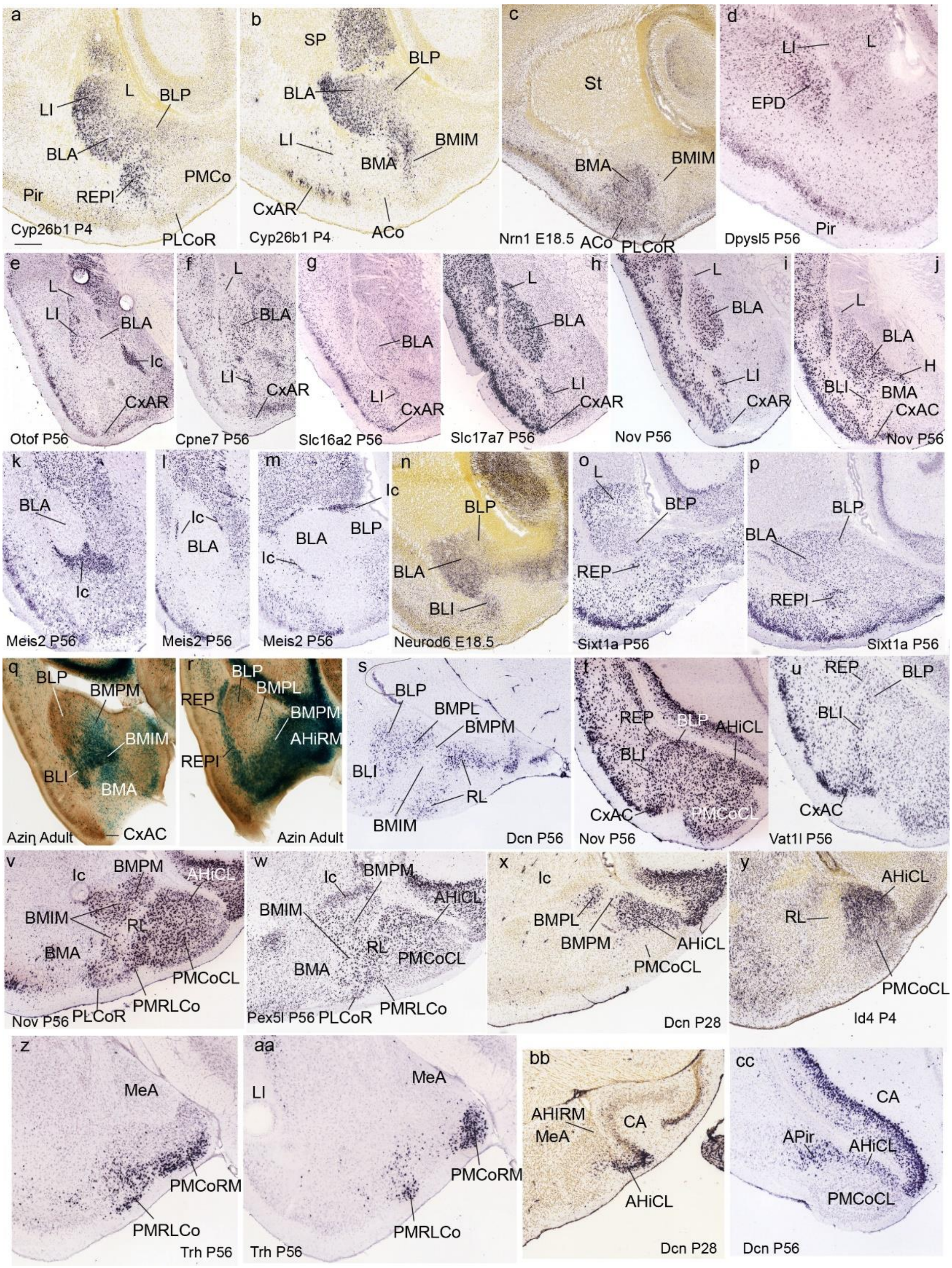
a
LI
L
BLP
BLA
Pir
REPI
PMCo
PLCoR
Cyp26b1 P4
b
SP
BLP
BLA
LI
BMIM
BMA
CxAR
ACo
Cyp26b1 P4
c
St
BMA
BMIM
ACo
PLCoR
Nrn1 E18.5
d
LI
L
EPD
Pir
Dpysl5 P56
e
L
LI
BLA
Ic
CxAR
Otof P56
f
L
BLA
LI
CxAR
Cpne7 P56
g
BLA
LI
CxAR
Slc16a2 P56
h
L
BLA
LI
CxAR
Slc17a7 P56
i
L
BLA
LI
CxAR
Nov P56
j
L
BLA
H
BLI
BMA
CxAC
Nov P56
k
BLA
Ic
Meis2 P56
l
Ic
BLA
Meis2 P56
m
Ic
BLA
BLP
Ic
Meis2 P56
n
BLP
BLA
BLI
Neurod6 E18.5
o
L
BLP
REP
Sixt1a P56
p
BLP
BLA
REPI
Sixt1a P56
q
BLP
BMPM
BMIM
BLI
BMA
CxAC
Azin Adult
r
BLP
REP
BMPL
BMPM
AHiRM
REPI
Azin Adult
s
BLP
BMPL
BMPM
BLI
RL
BMIM
Dcn P56
t
REP
BLP
AHiCL
BLI
CxAC
PMCoCL
Nov P56
u
REP
BLP
BLI
CxAC
Vat1l P56
v
Ic
BMPM
AHiCL
BMIM
RL
BMA
PMCoCL
PLCoR
PMRLCo
Nov P56
w
Ic
BMPM
BMIM
AHiCL
RL
PMCoCL
BMA
Pex5l P56
PLCoR
PMRLCo
x
Ic
BMPL
BMPM
AHiCL
PMCoCL
Dcn P28
y
AHiCL
RL
PMCoCL
Id4 P4
z
MeA
PMCoRM
PMRLCo
Trh P56
aa
LI
MeA
PMCoRM
PMRLCo
Trh P56
bb
AHIRM
MeA
CA
AHiCL
Dcn P28
cc
CA
APir
AHiCL
PMCoCL
Dcn P56

Suppl. Fig.1 Sample images downloaded from Allen Developmental Mouse Brain Atlas. These panels show a gallery of selected adult gene expression patterns in the pallial amygdala (largely at P56) serving to illustrate the types of patterns which we classified as given amygdalar structures in our Tables 1-5 and Suppl.Table 1. The mapped gene and specific mouse stage are indicated in every figure. (**a-d**, **m-p**, **v-y**, **bb**) are sagittal sections, with rostral oriented to the left and dorsal up, whereas (**e-l**, **q-u**, **z**, **aa**, **cc**) are standard coronal sections with the midline oriented to the right. (**q, r**) show AZIN2-LacZ material cut in the amygdalar radial plane; (**q)** shows the place where a thin AZIN2-positive part of BMPM reaches up to the BPL, and thus covers dorsally BMPL; compare with Fig.6a. (**r**) Shows another example of the radial continuity of the REP complex up to superficial levels, coursing lateral to BPL. Scale bar represent 400 μm.

Suppl. Fig.2

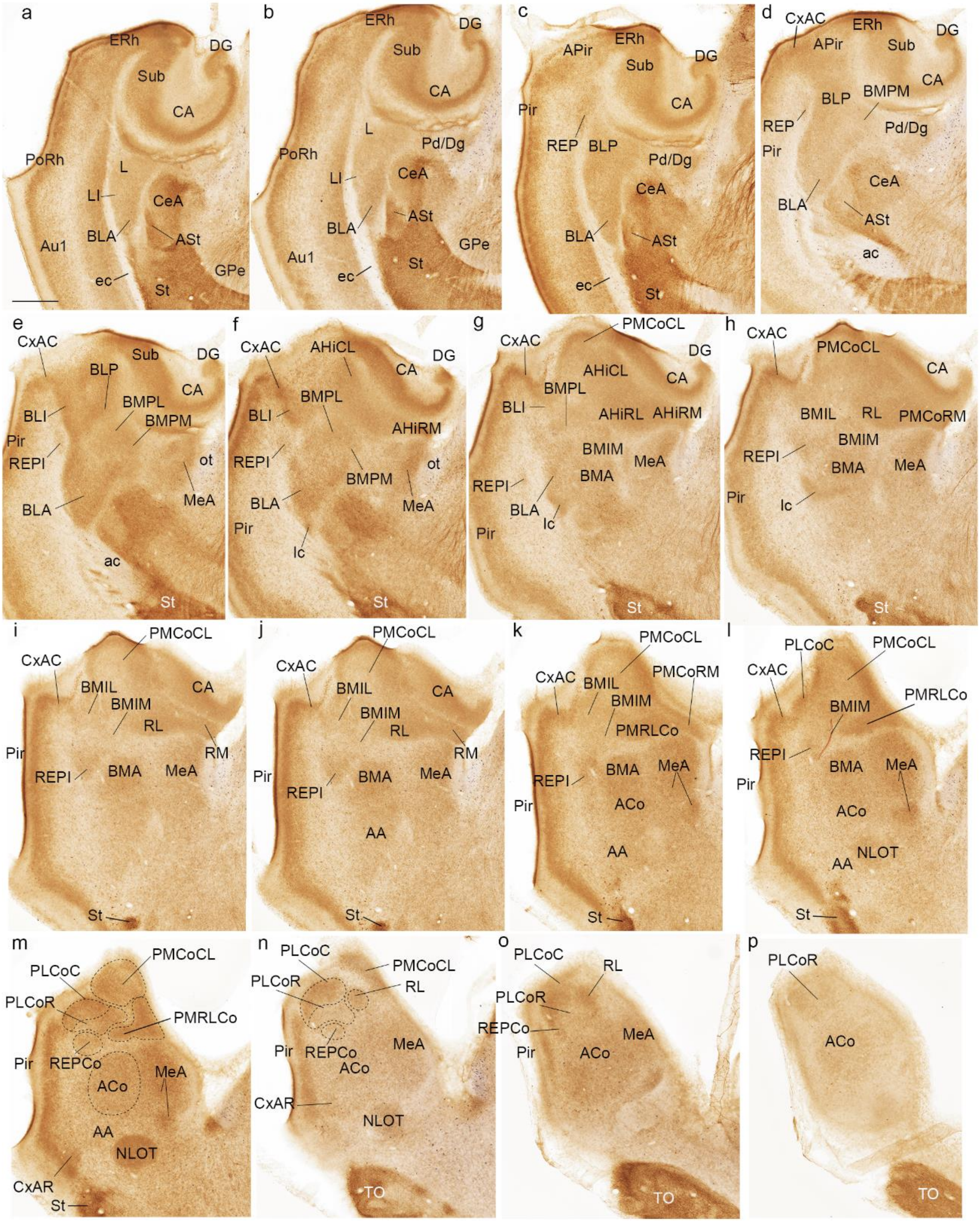

Suppl. Fig.2 Summary mapping of amygdalar radial units and their subpial superficial centers. We present here a continuous dorsoventral series of horizontal sections through the adult mouse amygdalar region which serendipituously happens to display different background densities which help to identify diverse amygdalar components. The pattern

that emerges distinctly corroborates our model. The material in question is an adult series from our collection which had been reacted for *Nkx2.2* ISH; the blue ISH signal only appears in these images as tiny labelled oligodendrocytes in the major fiber tracts (e.g., the optic tract), and are irrelevant for our purposes. The series was further weakly counterstained with TH immunoreaction, whose brown result can be seen best at the olfactory tuberculum, striatum and central amygdala neuropiles (TO, St, CeA), as well as intercalate nuclei (Ic). The remaining staining is due to background of the immunoreaction, which happens to make discernible a number of amygdalar elements. (**a-d**) these dorsal levels show appearance of the L, LI, BLP and BLA nuclei, jointly with the REP. Note clearcut boundaries of the pallial amygdala with both laterally placed cortex and rostromedially placed subpallial elements (St, ASt, CeA, MeA), as well as the amygdalar part of the lateral ventricle, separating the hippocampal complex. (**e-h**) These levels show the transition of BLP/BLA via BLI into CxAC (**e-g**), adjacent REP band, appearance of BMPL and BMPM nuclei and related BMIL and BMIM intermediate elements, emergence of the BMA nucleus under the medial part of BLA, extending into ACo (**i-l**), and begin of the AHiRM just under the end of the ventricle and its ulterior extension into AHiRL and AHiCL, before reaching underlying PMCo intermediate and superficial levels. (**i-l**) These levels bring us closer to the brain surface, without yet reaching it; we detect the BMIL/BMIM elements, which converge jointly with the REP and the RL part of PMCo towards the neighborhood of the PLCo.

(**m-p**) At these levels the CL and RM parts of PMCo start disappearing, as we start to enter into the PLCo (this reveals the section plane was not exactly horizontal). At the brain surface we recognize the NLOT nucleus, as well as the CxAR, the REPCo, the PLCoR, PLCoC and the PMRLCo, in characteristic respective positions, according to the model. Scale bar represents 900 µm.

Suppl.Table1.

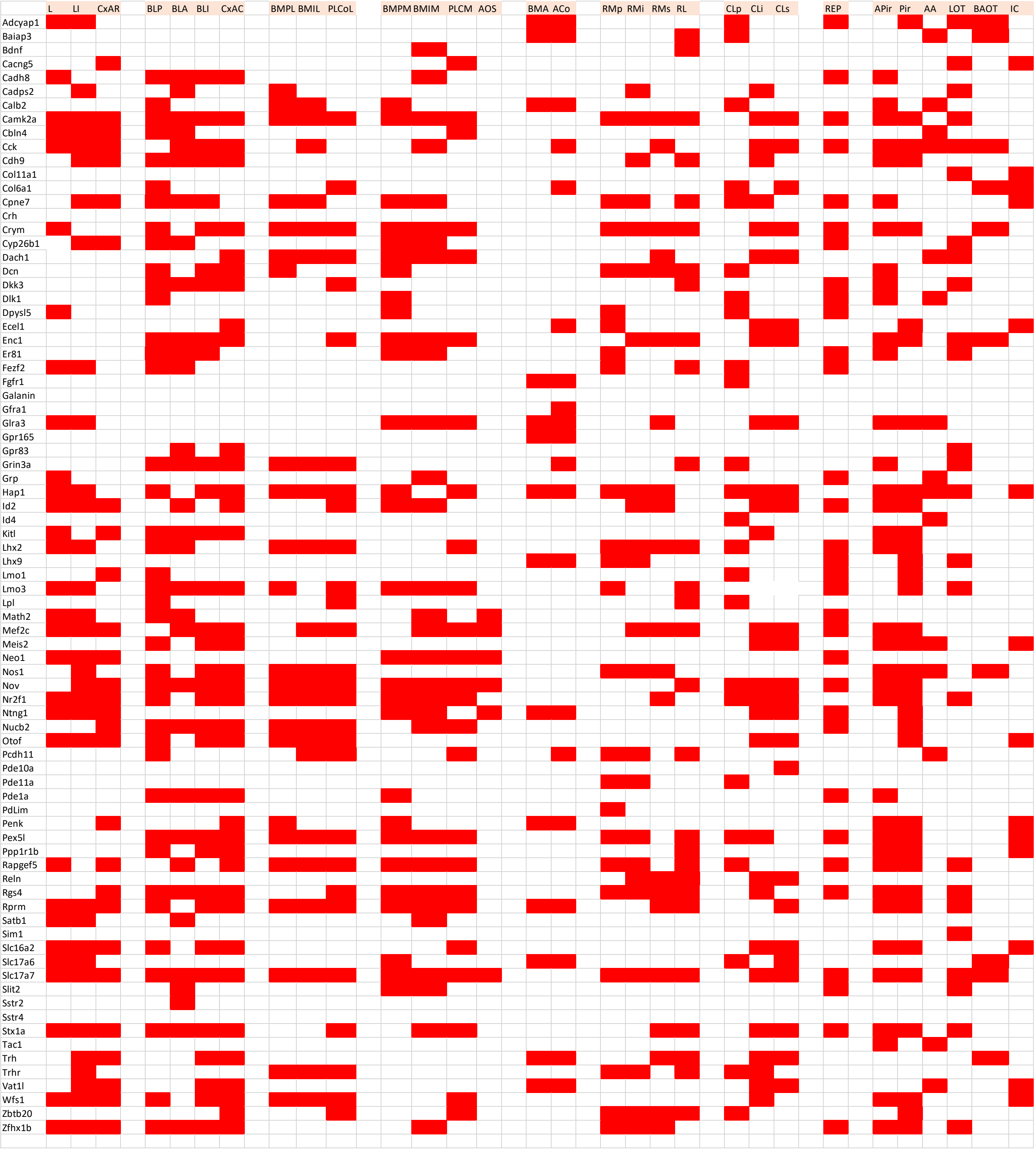

Suppl.Table1. Expression mapping of 81 gene markers from the Allen Developing Mouse Brain Atlas according to the radial amygdala model. The anatomic structures are grouped according to radial units or subunits, as well as according to the strata they occupy; the names appear above, some of them abbreviated due to space considerations (see below). Red color entries code for positive expression at the specified loci. Abbreviations used for the habitual name tags: RMp instead of AHiRM; RMi instead of PMCoiRM; RMs instead of PMCoRM; RL instead of AHiRL and PMCoRL; CLp instead of AHiCL; CLi instead of PMCoCLi; CLs instead of PMCoCLs.
